# Non-pathogenic *Escherichia coli* acquires virulence by mutating a growth-essential LPS transporter

**DOI:** 10.1371/journal.ppat.1008469

**Published:** 2020-04-23

**Authors:** Chikara Kaito, Hirono Yoshikai, Ai Wakamatsu, Atsushi Miyashita, Yasuhiko Matsumoto, Tomoko Fujiyuki, Masaru Kato, Yoshitoshi Ogura, Tetsuya Hayashi, Takao Isogai, Kazuhisa Sekimizu

**Affiliations:** 1 Graduate School of Medicine, Dentistry, and Pharmaceutical Sciences, Okayama University, Kita-ku, Okayama, Japan; 2 Graduate School of Pharmaceutical Sciences, The University of Tokyo, Bunkyo-ku, Tokyo, Japan; 3 Japan Biological Informatics Consortium (JBIC), Koto-ku, Tokyo, Japan; 4 Department of Microbiology, Meiji Pharmaceutical University, Kiyose, Tokyo, Japan; 5 The Institute of Medical Science, The University of Tokyo, Minato-ku, Tokyo, Japan; 6 Devision of Bioanalytical Chemistry, School of Pharmacy, Showa University, Shinagawa-ku, Tokyo, Japan; 7 Department of Bacteriology, Faculty of Medical Sciences, Kyushu University, Fukuoka, Japan; 8 Translational Research Center, Fukushima Medical University, Fukushima, Japan; 9 Institute of Medical Mycology, Teikyo University, Hachioji, Tokyo, Japan; Channing Laboratory, Brigham and Women's Hospital, UNITED STATES

## Abstract

The molecular mechanisms that allow pathogenic bacteria to infect animals have been intensively studied. On the other hand, the molecular mechanisms by which bacteria acquire virulence functions are not fully understood. In the present study, we experimentally evaluated the evolution of a non-pathogenic strain of *Escherichia coli* in a silkworm infection model and obtained pathogenic mutant strains. As one cause of the high virulence properties of *E*. *coli* mutants, we identified amino acid substitutions in LptD (G580S) and LptE (T95I) constituting the lipopolysaccharide (LPS) transporter, which translocates LPS from the inner to the outer membrane and is essential for *E*. *coli* growth. The growth of the LptD and LptE mutants obtained in this study was indistinguishable from that of the parent strain. The LptD and LptE mutants exhibited increased secretion of outer membrane vesicles containing LPS and resistance against various antibiotics, antimicrobial peptides, and host complement. *In vivo* cross-linking studies revealed that the conformation of the LptD-LptE complex was altered in the LptD and LptE mutants. Furthermore, several clinical isolates of *E*. *coli* carried amino acid substitutions of LptD and LptE that conferred resistance against antimicrobial substances. This study demonstrated an experimental evolution of bacterial virulence properties in an animal infection model and identified functional alterations of the growth-essential LPS transporter that led to high bacterial virulence by conferring resistance against antimicrobial substances. These findings suggest that non-pathogenic bacteria can gain virulence traits by changing the functions of essential genes, and provide new insight to bacterial evolution in a host environment.

## Author summary

Pathogenic bacteria developed their virulence properties by changing the functions of various genes after the emergence of the host animals on earth. The types of gene function alterations that confer bacterial virulence properties, however, have remained unclear. We utilized a silkworm infection model to perform an experimental evolution of bacterial virulence activity. From a non-pathogenic strain of *Escherichia coli*, we obtained a mutant strain that exhibited 500-fold higher virulence than the original strain and identified mutations of the lipopolysaccharide (LPS) transporter, which translocates LPS onto the bacterial surface, as one cause of the high virulence. The mutations changed the structure of the LPS transporter, increased the secretion of outer membrane vesicles, and enabled bacterial survival in the presence of host antimicrobial substances. This mechanism to gain high virulence occurs naturally, as several *E*. *coli* clinical isolates carried mutations of the LPS transporter that confer resistance against antimicrobial substances. Our study unveiled a novel mechanism by which bacteria increase their virulence through modifying their gene function.

## Introduction

Uncovering the difference between pathogenic bacteria and non-pathogenic bacteria is important toward understanding the mechanisms of bacterial virulence and developing novel medicines against bacterial infectious diseases. Although previous studies using gene-knockout methods or comparative genomics have revealed many bacterial genes required for virulence including toxin genes encoded on mobile genetic elements, how pathogenic bacteria acquire virulence traits, especially the gene mutations that confer bacterial virulence, during evolution has remained unclear. Experimental evolution is a novel, recently developed system to uncover the molecular mechanisms by which cells acquire various functions, such as temperature resistance [1] and antibiotic resistance [2]. Experimental evolution of virulence properties was evaluated *in vitro* with viruses against eukaryotic cells [3], bacteriophages against bacteria [4, 5], and bacteria against macrophages [2, 6]. To our knowledge, there are no *in vivo* studies of experimental evolution using an infectious agent against animals. In the present study, we performed an experimental evolution of a non-pathogenic laboratory strain of *Escherichia coli* into a pathogenic strain in the silkworm infection model, which is highly useful for infection experiments [7, 8]. We successfully obtained bacterial mutants with 500-fold higher virulence than the original strain and found that amino acid substitutions of the lipopolysaccharide (LPS) transporter increase *E*. *coli* virulence.

LPS are glycolipids constituting the outer leaflet of Gram-negative bacteria that defend bacteria against various extracellular stresses [9, 10]. LPS are also contained in outer membrane vesicles (OMVs), which many Gram-negative bacteria secrete to the extracellular milieu [11]. OMVs have pleiotropic functions such as defending against extracellular stresses, transporting bacterial toxins to target cells, and acting as a vector for transmitting genetic information to other bacterial cells [12, 13]. LPS is synthesized at the inner membrane and transported to the outer membrane by an LPS transporter comprising seven subunits, LptA, LptB, LptC, LptD, LptE, LptF, and LptG [14]. All seven subunits are essential for *E*. *coli* growth [14–16]. Mutations of the LPS transporter sensitize *E*. *coli* against many antibiotics [17]. The role of the LPS transporter in bacterial virulence has not been revealed.

In the present study, we found that amino acid substitutions of the LPS transporter increased the amount of OMVs and conferred bacterial resistance against host-derived antimicrobial substances. Furthermore, we found amino acid substitutions of the LPS transporter in clinical isolates of *E*. *coli* that contributed to resistance against antimicrobial substances. The present study is the first to demonstrate that amino acid substitutions of the growth-essential LPS transporter increase bacterial virulence properties.

## Results

### Amino acid substitutions of LptD and LptE increase *E*. *coli* virulence against silkworms

**Fig 1:**
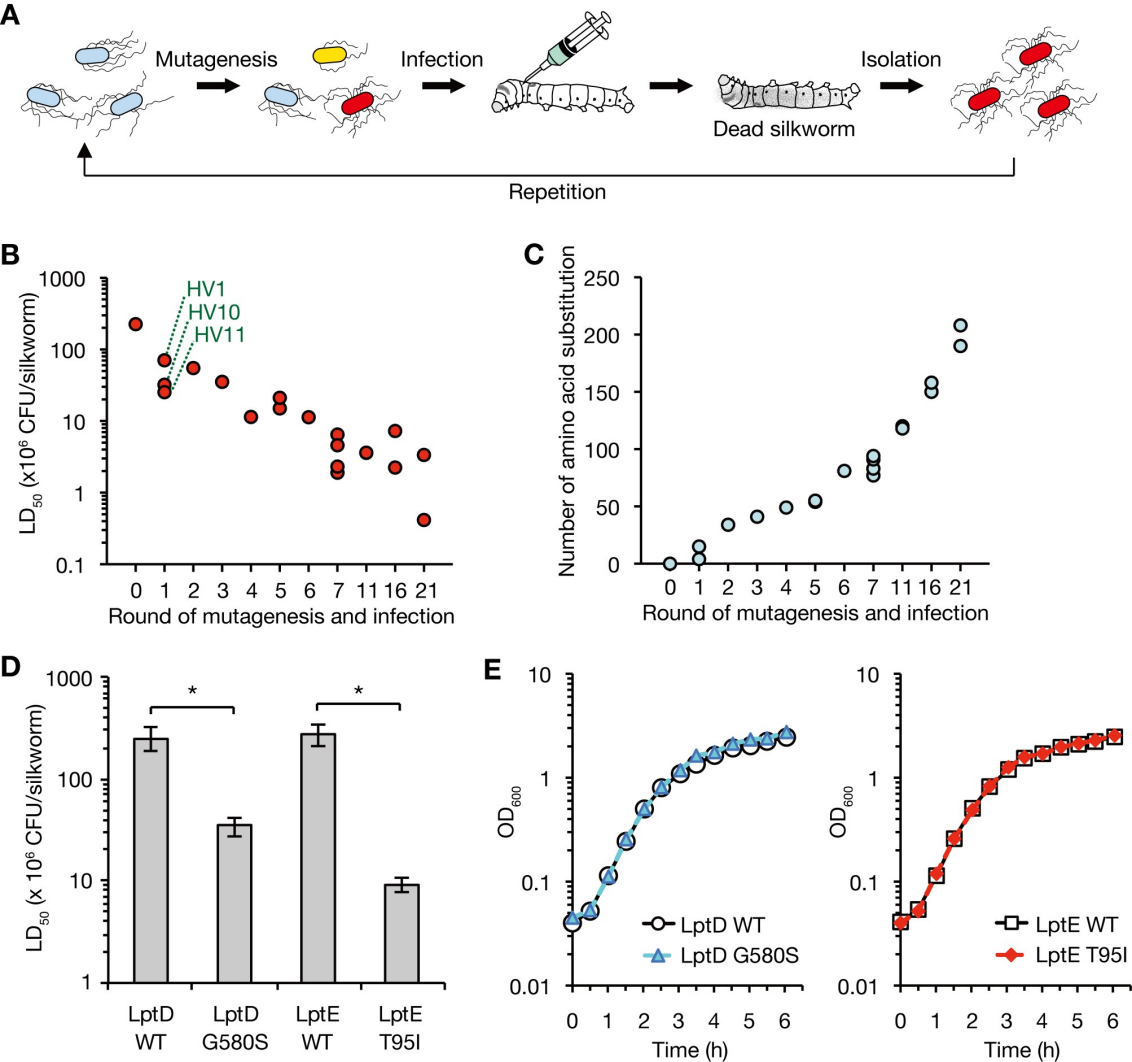
Isolation of *E*. *coli* mutants with high virulence by experimental evolution. **(A)** A schematic representation of experimental evolution utilizing the silkworm infection model. *E*. *coli* strain treated with a mutagen was injected into silkworm. After the silkworm died, *E*. *coli* strains were isolated from the dead silkworm hemolymph. The mutagenesis and infection were repeated for 21 cycles. **(B)** An *E*. *coli* single colony isolated by the experimental evolution was cultured overnight, serially diluted, and injected into silkworms. Silkworm survival was determined at 48 h after the injection and the LD_50_ was determined by logistic regression. The horizontal axis represents the number of times the mutagenesis and infection experiments were performed. Each colony isolated from a dead silkworm that appeared different from the others was examined for its killing activity. The three symbols marked by green dotted lines represent the HV1, HV10, and HV11 strains. **(C)** *E*. *coli* strains isolated in the experimental evolution were subjected to whole genome sequencing and the number of amino acid substitutions is presented. The horizontal axis and strains are the same as in B. **(D)** Overnight cultured bacterial cells of the LptD WT, LptD G580S, LptE WT, and LptE T95I strains were serially diluted and injected into silkworms. Silkworm survival was determined at 48 h after the injection and the LD_50_ was determined by logistic regression. Data shown are the mean ± standard errors from three independent experiments. The asterisk represents a p value less than 0.05 (Student’s *t* test). **(E)** *E*. *coli* strains of LptD WT, LptD G580S, LptE WT, and LptE T95I were aerobically cultured in LB broth at 37˚C. The vertical axis represents the OD_600_ of bacterial culture, and the horizontal axis represents the culture time.

We repeatedly treated a laboratory strain of *E*. *coli* with a mutagen and subsequently infected silkworms to obtain *E*. *coli* mutants with increased killing activity against the silkworms (**Fig 1A**). As we performed more rounds of mutagenesis and infection, the median lethal dose (LD_50_) of the isolated strain in silkworms decreased (**Fig 1B**). After 21 rounds of mutagenesis and infection, we obtained a mutant strain with a 500-fold lower LD_50_ than that of the parent strain. Whole genome sequencing revealed that gene mutations causing amino acid substitutions increased in the mutant strains that exhibited increased killing activity against silkworms (**Fig 1C**). These results suggest that the accumulation of gene mutations increases the *E*. *coli* virulence against silkworms.

Three mutant strains, named HV1, HV10, and HV11, which were obtained by a single round of mutagenesis and infection, had a lower LD_50_ than that of the parent strain (**Fig 1B**) and had 4, 15, and 15 gene mutations, respectively, causing amino acid substitutions (**S1 Table**). Among the mutated gene products, the LPS transporter was identified in all three mutants; HV1 had an LptD G580S mutation, and HV10 and HV11 had an LptE T95I mutation. We examined whether the amino acid substitutions of the LPS transporter led to the high virulence of *E*. *coli* against silkworms by constructing LptD and LptE mutants. A transposon marker was inserted near the *lptD* in the HV1 strain and *lptE* in the HV11 strain. The *lptD* mutation in HV1 or the *lptE* mutation in HV11 was transferred to the parent strain by phage transduction using the transposon marker, resulting in the LptD G580S mutant or the LptE T95I mutant, respectively. The LptD G580S mutant had a lower LD_50_ than the isogenic parent strain carrying the same transposon marker (LptD WT) (**Fig 1D**). The LptE T95I mutant had a lower LD_50_ than the isogenic parent strain carrying the same transposon marker (LptE WT) (**Fig 1D**). These findings suggest that LptD G580S and LptE T95I conferred high virulence properties of *E*. *coli* against silkworms.

LptD and LptE are essential for *E*. *coli* growth. We examined whether the LptD G580S mutant and LptE T95I mutant impair growth. The growth of the LptD G580S mutant and LptE T95I mutant was indistinguishable from that of their respective parent strains in nutrient broth (**Fig 1E**). The results suggest that LptD G580S and LptE T95I do not affect the growth capability of *E*. *coli*.

### Amino acid substitutions of LptD and LptE confer resistance against host humoral immunity, antibiotics, and organic solvent

**Fig 2:**
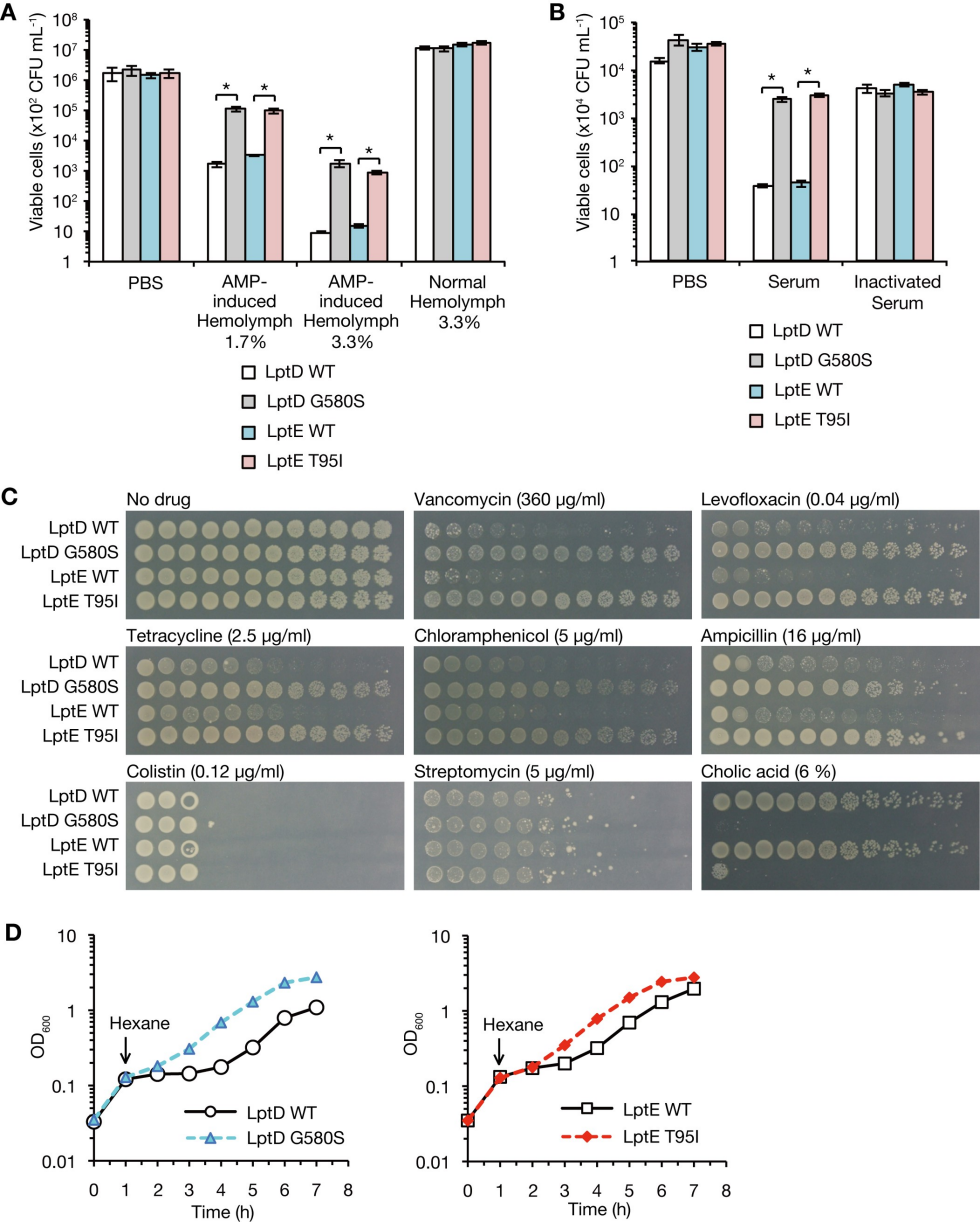
The LptD and LptE mutants are resistant to various antimicrobial substances. **(A)** The LptD WT, LptD G580S, LptE WT, and LptE T95I strains were incubated at 37˚C for 30 min in PBS, 1.7% or 3.3% silkworm hemolymph in which antimicrobial peptides were induced (AMP-induced Hemolymph), or 3.3% silkworm hemolymph in which antimicrobial peptides was not induced (Normal Hemolymph). After incubation, the number of live bacterial cells was determined. Data shown are the mean ± standard errors from two independent experiments performed in triplicate. The asterisk represents a p value less than 0.05 (Student’s *t* test). **(B)** The LptD WT, LptD G580S, LptE WT, and LptE T95I strains were incubated at 37˚C for 45 min in PBS, porcine serum (Serum), or heat-treated porcine serum (Inactivated Serum). After incubation, the number of live bacterial cells was determined. Data shown are the mean ± standard errors from two independent experiments performed in triplicate. The asterisk represents a p value less than 0.05 (Student’s *t* test). **(C)** Overnight cultures of LptD WT, LptD G580S, LptE WT, and LptE T95I strains were 5-fold serially diluted, spotted onto LB agar plates supplemented without or with vancomycin, levofloxacin, tetracycline, chloramphenicol, ampicillin, colistin, streptomycin, or cholic acid, and incubated at 37˚C. **(D)** The LptD WT, LptD G580S, LptE WT, and LptE T95I strains were aerobically cultured at 37˚C in LB broth, and n-hexane was added to the culture at 1 h after the incubation. The OD_600_ of the bacterial cultures were measured every 1 h.

Based on the finding that the LptD G580S and LptE T95I mutants exhibited increased virulence against silkworms, we examined whether these mutants are resistant to antimicrobial peptides that constitute the innate immune system of silkworms. In silkworm hemolymph in which antimicrobial peptides were induced, the viable cell numbers of the LptD G580S and LptE T95I mutants were more than 30-fold that of the respective parent strain (**Fig 2A**). In contrast, there was no difference in the viable cell number between the mutants and parent strains in silkworm hemolymph in which antimicrobial peptides were not induced, or in phosphate-buffered saline (PBS) (**Fig 2A**). These findings suggest that the LptD G580S and LptE T95I mutants are resistant to silkworm antimicrobial peptides.

To determine whether the LptD G580S and LptE T95I mutants are resistant to the mammalian immune system, we examined bacterial resistance against swine serum complement. The number of viable cells of the LptD G580S and LptE T95I mutants was more than 60-fold that of the respective parent strain in swine serum (**Fig 2B**). No difference in the viable cell numbers was observed in heat-treated swine serum in which complements were inactivated, or in PBS (**Fig 2B**). These results suggest that the LptD G580S and LptE T95I mutants were resistant to mammalian complement.

LptD and LptE mutations sensitize *E*. *coli* to vancomycin and cholic acid by abolishing the outer membrane barrier function [17]. In addition, the LptD mutation sensitizes *E*. *coli* to an organic solvent, n-hexane [18]. On this basis, together with our findings that the LptD G580S and LptE T95I mutants were resistant to host antimicrobial peptides and complement, we hypothesized that the LptD G580S and LptE T95I mutants are resistant to various antimicrobial substances, such as antibiotics, cholic acid, and an organic solvent. The LptD G580S and LptE T95I mutants exhibited better growth than their respective parent strains in the presence of vancomycin, levofloxacin, tetracycline, chloramphenicol, ampicillin, or colistin, although the growth difference was not observed in the presence of streptomycin (**Fig 2C**). In the presence of cholic acid, the LptD G580S and LptE T95I mutants exhibited growth defects compared with their respective parent strains (**Fig 2C**). In the presence of n-hexane, the LptD G580S and LptE T95I mutants grew faster than their respective parent strains (**Fig 2D**). These findings suggest that the LptD G580S and LptE T95I mutants are resistant to several antibiotics and n-hexane, but are sensitive to cholic acid.

To clarify whether the LptD G580S and LptE T95I mutants have increased virulence in a mammalian infection model, we examined their bacterial colonization efficiency in mouse intestine. The number of colonies recovered from mice feces did not differ between the LptD G580S and LptE T95I mutants and their respective parent strains (**S1A Fig**). The competition assay did not detect the difference of the colonization efficiency between the LptD G580S and LptE T95I mutants and their respective parent strains (**S1B Fig**). Therefore, the LptD G580S and LptE T95I mutants do not increase colonization ability in mouse intestine.

### LptD G580S and LptE T95I exhibit increased production of OMVs

**Fig 3:**
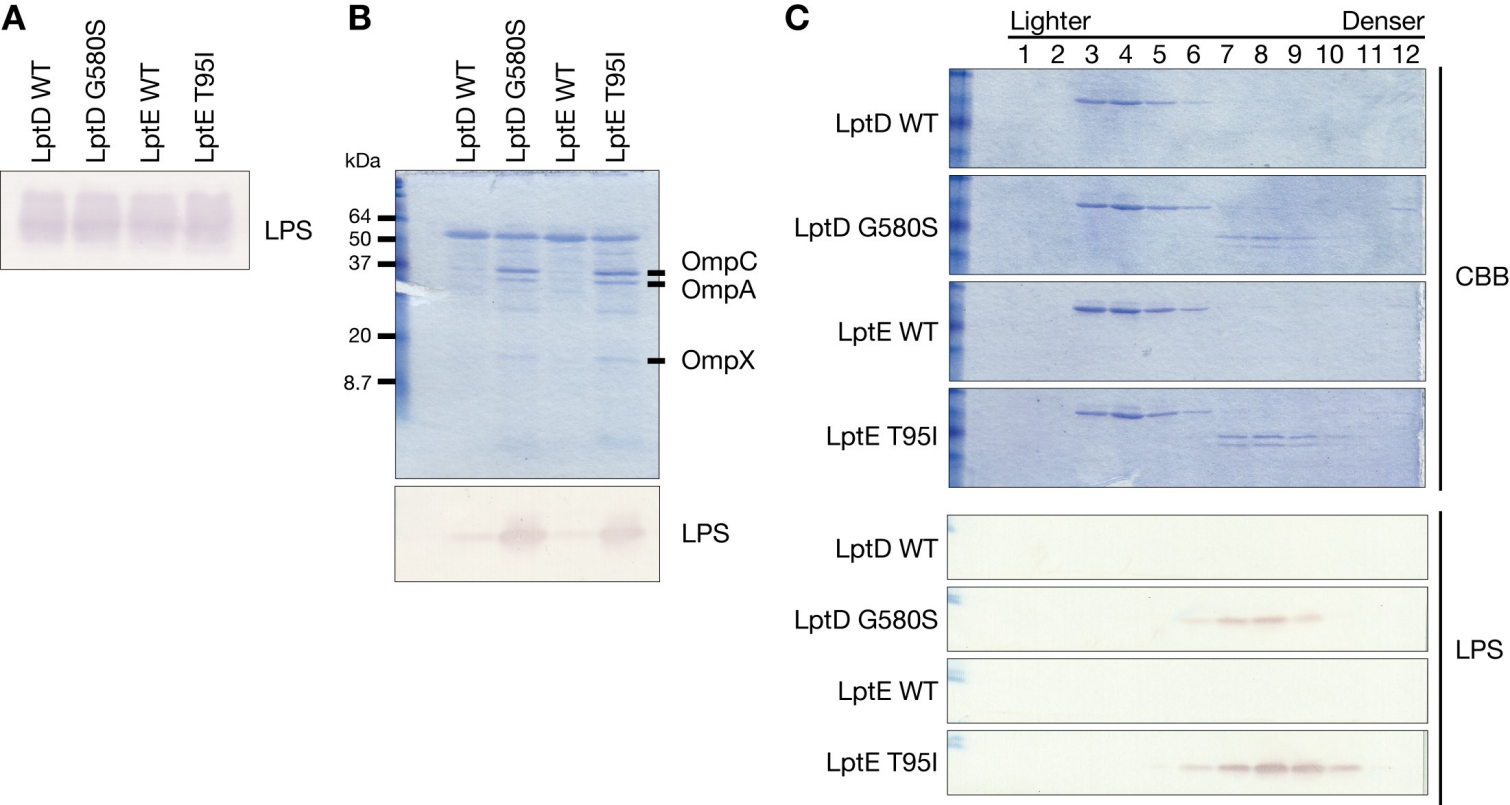
The LptD and LptE mutants increase production of OMVs. **(A)** Overnight cultured bacterial cells of the LptD WT, LptD G580S, LptE WT, and LptE T95I strains were lysed, electrophoresed in SDS-polyacrylamide gels, and subjected to Western blot analysis using an anti-LPS antibody. **(B)** Culture supernatants of the LptD WT, LptD G580S, LptE WT, and LptE T95I strains were ultracentrifuged and the precipitates were electrophoresed in SDS-polyacrylamide gels. The gels were stained by Coomassie Brilliant Blue (upper panel). Bands for OmpC, OmpA, and OmpX were identified by peptide mass fingerprinting analysis (**S2 Table**). The same samples were also subjected to Western blot analysis using an anti-LPS antibody (lower panel). **(C)** Culture supernatants of the LptD WT, LptD G580S, LptE WT, and LptE T95I strains were ultracentrifuged and the precipitates were further fractionated by density gradient centrifugation. The fractionated samples were electrophoresed in SDS-polyacrylamide gels and stained with Coomassie Brilliant Blue (upper panel). Also, the fractionated samples were subjected to Western blot analysis using an anti-LPS antibody (lower panel).

To clarify how the LptD G580S and LptE T95I mutants increase resistance against extracellular antimicrobial substances, we examined the amount of LPS in bacterial cells and in the OMVs. The amount of LPS in the bacterial cells did not differ between the LptD G580S and LptE T95I mutants and their respective parent strains (**Fig 3A**). In contrast, the amount of LPS in the OMV fraction was increased in the LptD G580S and LptE T95I mutants compared with that in their respective parent strains (**Fig 3B**). In addition, the outer membrane proteins OmpC, OmpA, and OmpX in the OMV fraction were increased in the LptD G580S and LptE T95I mutants compared with their respective parent strains (**Fig 3B, S2 Table**). To confirm the presence of these molecules in the OMVs, we further fractionated the OMV fraction by density gradient centrifugation. LPS, OmpC, and OmpA were detected in the same intermediate fractions (fr. 7–9) of the density gradient centrifugation, indicating that these molecules are present in the OMVs (**Fig 3C**). Measurement of the diameter by dynamic light-scattering assay revealed that the amount of OMVs with a diameter less than 100 nm was increased in the LptD G580S and LptE T95I mutants (**S2 Fig**). Taken together, these results suggest that OMVs are increased in the LptD G580S and LptE T95I mutants.

### LptD G580S and LptE T95I increase efflux activity against foreign chemicals

**Fig 4:**
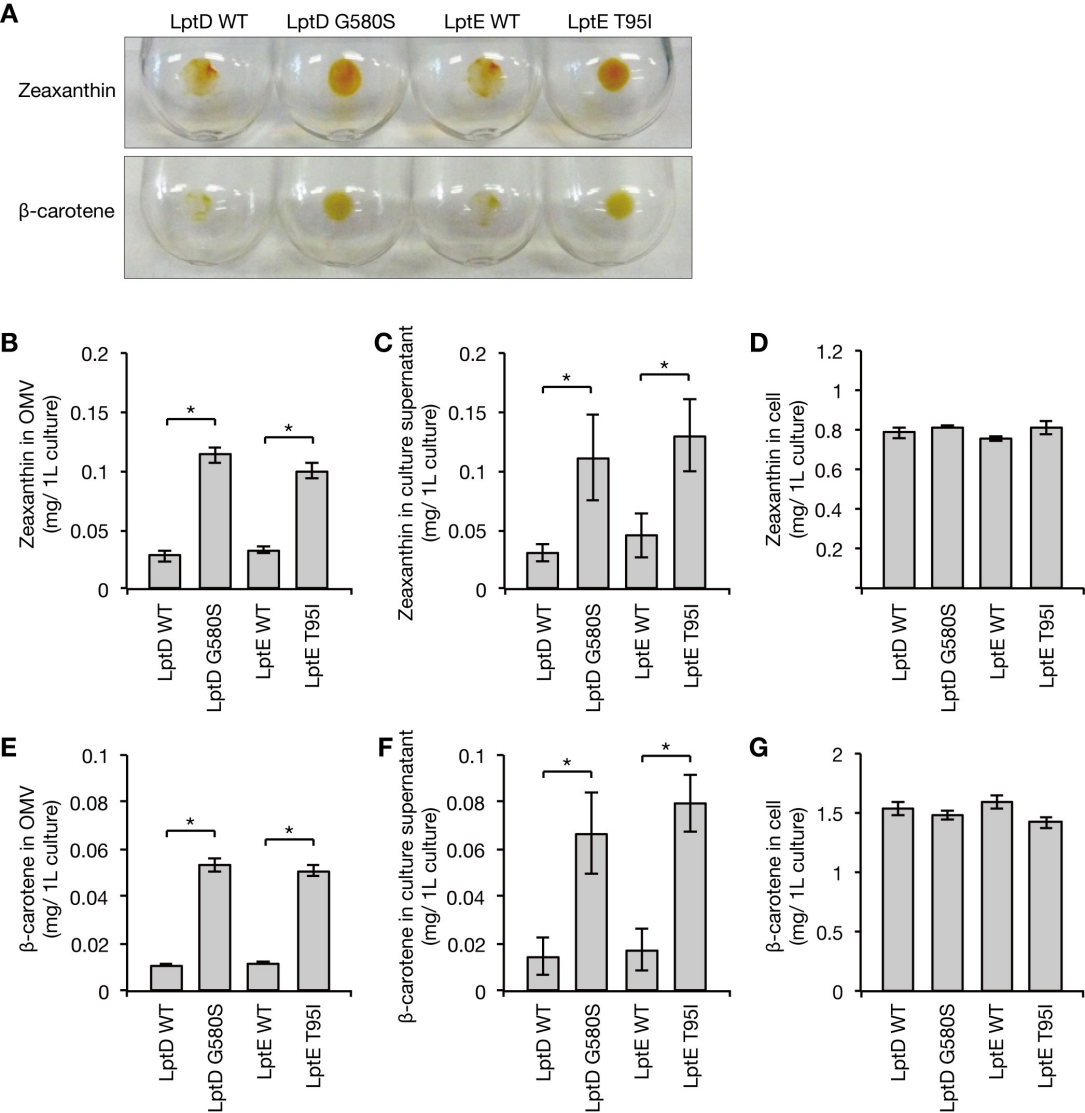
The LptD and LptE mutants extrude foreign chemicals together with OMVs. **(A)** The LptD WT, LptD G580S, LptE WT, and LptE T95I strains were transformed with plasmids to produce isoprenoids (zeaxanthin or β-carotene). The culture supernatants were ultracentrifuged for collection of OMVs. Images are the precipitates of ultracentrifugation. **(B)** The amounts of zeaxanthin in OMV fractions from the LptD WT, LptD G580S, LptE WT, and LptE T95I strains were measured. Data shown are means ± standard errors from three independent experiments. The asterisk represents a p value less than 0.05 (Student’s *t* test). **(C)** The amounts of zeaxanthin in the culture supernatants from the LptD WT, LptD G580S, LptE WT, and LptE T95I strains were measured. Data shown are means ± standard errors from three independent experiments. The asterisk represents a p value less than 0.05 (Student’s *t* test). **(D)** The amount of zeaxanthin in bacterial cells of the LptD WT, LptD G580S, LptE WT, and LptE T95I strains was measured. Data shown are means ± standard errors from three independent experiments. **(E)** The amount of β-carotene in OMV fractions from the LptD WT, LptD G580S, LptE WT, and LptE T95I strains was measured. Data shown are means ± standard errors from three independent experiments. The asterisk represents a p value less than 0.05 (Student’s *t* test). **(F)** The amounts of β-carotene in the culture supernatants from the LptD WT, LptD G580S, LptE WT, and LptE T95I strains were measured. Data shown are means ± standard errors from three independent experiments. The asterisk represents a p value less than 0.05 (Student’s *t* test). **(G)** The amount of β-carotene in bacterial cells of the LptD WT, LptD G580S, LptE WT, and LptE T95I strains was measured. Data shown are means ± standard errors from three independent experiments.

OMVs adsorb various antimicrobial substances and thus contribute to bacterial resistance against antimicrobial substances [19]. Also, overproduction of the ABC transporter MsbA, which translocates LPS across the inner membrane, increases the efflux activity of isoprenoids that are artificially synthesized in bacterial cells [20]. Because the LptD G580S and LptE T95I mutants were resistant to several antimicrobial substances, and exhibited increased OMV production, we considered the possibility that increased OMV production causes bacterial resistance to antimicrobial substances by increasing efflux activity against foreign chemicals. To address this point, we measured the amounts of the isoprenoids, zeaxanthin and ß-carotene, which were artificially produced in bacterial cells and secreted to the extracellular milieu together with the OMVs. The LptD G580S and LptE T95I mutants secreted greater amounts of zeaxanthin together with the OMVs than their respective parent strains (**Fig 4A and 4B**). Consistent with this observation, the amount of zeaxanthin in the culture supernatant was increased in the LptD G580S and LptE T95I mutants compared with that in their respective parent strains (**Fig 4C**). In contrast, the amount of zeaxanthin in the bacterial cells did not differ between the LptD G580S and LptE T95I mutants and their respective parent strains (**Fig 4D**). Furthermore, the amounts of ß-carotene together with the OMVs or in the culture supernatant were increased in the LptD G580S and LptE T95I mutants compared with those in their respective parent strains, but the amount of ß-carotene was not different in the bacterial cells (**Fig 4A, 4E, 4F and 4G**). These findings suggest that the LptD G580S and LptE T95I mutants increase the efflux activity of foreign chemicals.

### LptD G580S and LptE T95I affect the conformation of the LptD-LptE complex

**Fig 5:**
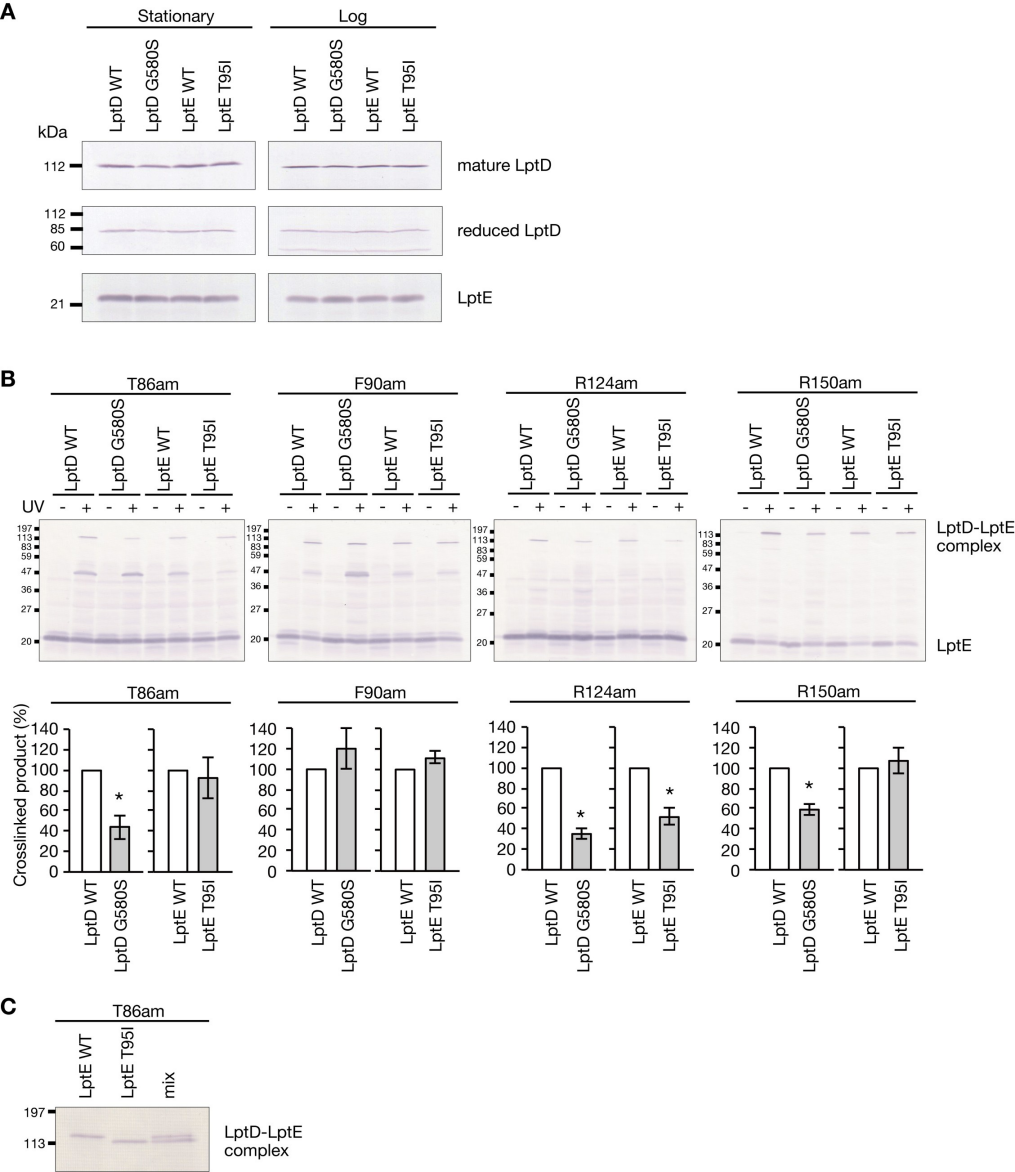
*In vivo* cross-linking analysis of the LptD-LptE complex in the LptD and LptE mutants. **(A)** Bacterial cells of the LptD WT, LptD G580S, LptE WT, and LptE T95I strains at a stationary phase or logarithmic growth phase were lysed and subjected to Western blot analysis using an anti-LptD antibody or anti-LptE antibody. **(B)** The LptD WT, LptD G580S, LptE WT, and LptE T95I strains carrying the *lptE* amber mutations (T86am, F90am, R124am, and R150am) were cultured in the presence of *p*BPA. Each strain expressing *p*BPA-substituted LptE was irradiated with UV light or not irradiated, and subjected to Western blot analysis using anti-LptE antibody. Lower graphs indicate the relative band intensity of the LptD-LptE complex in the LptD or LptE mutants compared with that in the parent strain. Data shown are means ± standard errors from three independent experiments. The asterisk represents a p value less than 0.05 (Student’s *t* test). **(C)** UV-irradiated samples of the LptE WT and LptE T95I strains expressing LptE T86am were mixed and electrophoresed (right lane). The gel was subjected to Western blot analysis using anti-LptE antibody.

To understand the effects of LptD G580S and LptE T95I mutations on the molecular function of LptD and LptE, we first examined the amount of LptD and LptE by Western blot analysis. Immature LptD is changed to mature LptD by disulfide bond formation [21]. Sample treatment with β-mercaptoethanol produces a reduced LptD band attributed to both mature LptD and immature LptD, whereas the sample treatment without β-mercaptoethanol produces a band corresponding to the mature LptD [21]. The amounts of the mature LptD and the reduced LptD were not different between the LptD G580S and LptE T95I mutants and the respective parent strains in both logarithmic and stationary phases (**Fig 5A**). In addition, the amount of LptE did not differ between the LptD G580S and LptE T95I mutants and the respective parent strains in both logarithmic and stationary phases (**Fig 5A**). These results suggest that the LptD G580S and LptE T95I mutations do not affect the amount of LptD and LptE.

We then hypothesized that the molecular functions of LptD and LptE are affected by the LptD G580S and LptE T95I mutations. LptD and LptE form a complex on the outer membrane and constitute a final exit tunnel for LPS. We examined whether the LptD-LptE complex structure was changed in the LptD G580S and LptE T95I mutants. LptD and LptE interact with each other at several sites, which can be detected by *in vivo* cross-linking [22]. We introduced an amber mutation into chromosomal *lptE* in the presence of the tRNA synthetase/tRNA pair corresponding to the amber codon, and obtained *E*. *coli* strains in which T86, F90, R124, or R150 of endogenous LptE was replaced with a non-natural amino acid, L-4-benzoyl phenyl alanine (*p*BPA). Consistent with a previous report [22], both anti-LptE and anti-LptD antibodies detected LptD-LptE complex bands in a UV-irradiation dependent manner in all four *lptE* amber strains (**Fig 5B and S3 Fig**). In the LptD G580S mutant, band intensities of the LptD-LptE complexes, in which LptE residues T86, R124, or R150 were replaced with *p*BPA, were decreased compared with the LptD WT strain (**Fig 5B**). In the LptE T95I mutant, band intensity of the LptD-LptE complex, in which R124 was replaced with *p*BPA, was decreased compared with the LptE WT strain (**Fig 5B**). Additionally, the band of the LptD-LptE complex, in which T86 was replaced with *p*BPA, migrated faster in the LptE T95I mutant than in the LptE WT strain (**Fig 5C**). These results suggest that LptD G580S and LptE T95I change the structure of the LptD-LptE complex.

### Suppressor mutations against LptD G580S and LptE T95I mutations decrease LptD expression

**Fig 6:**
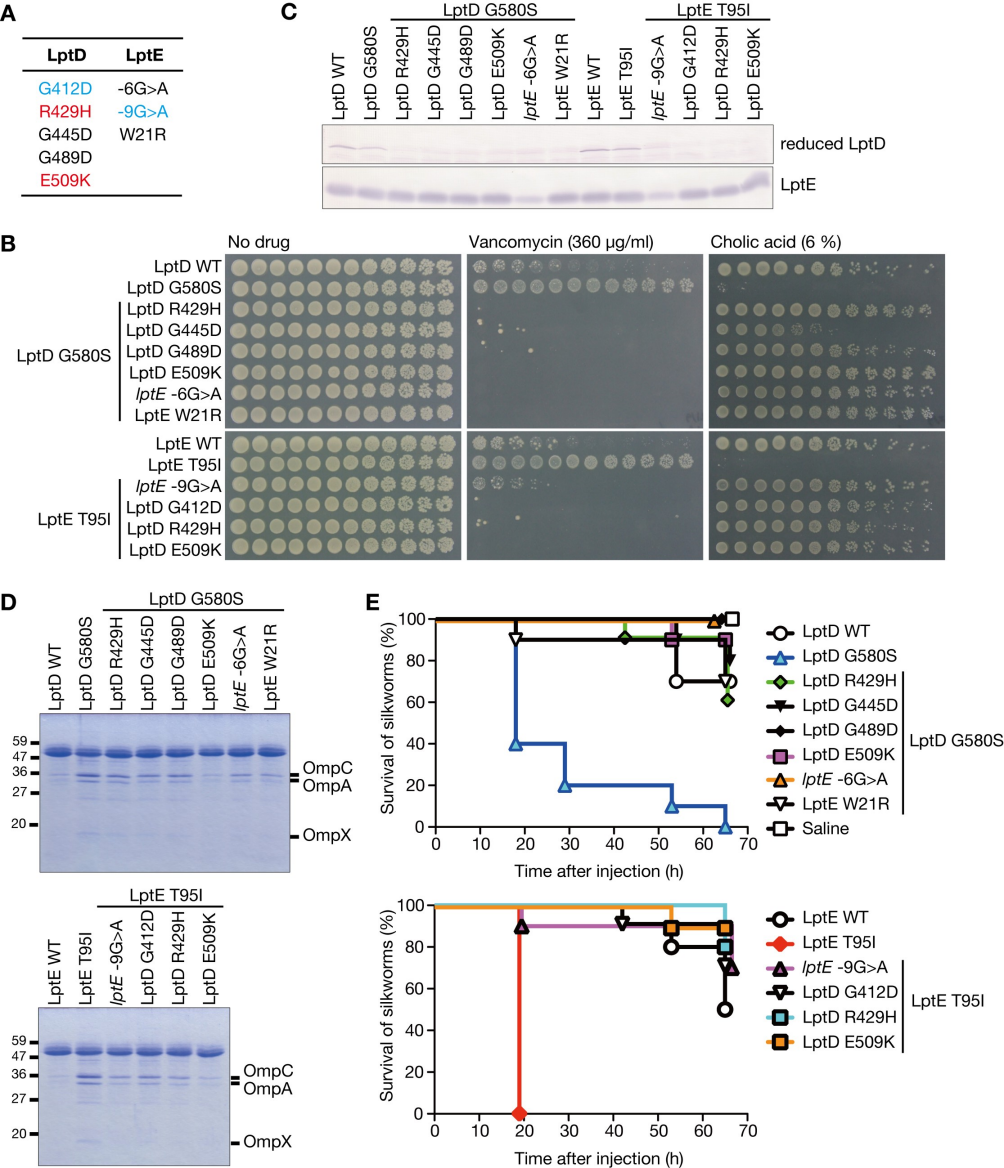
Genetic analysis of suppressors against the LptD and LptE mutations. **(A)** LptD and LptE mutations in the mutants resistant to cholic acid that originated from the LptD G580S and LptE T95I mutants are presented. Mutations colored in black, cyan, or red were found in the mutants originating from the LptD G580S mutant, the LptE T95I mutant, or both mutants, respectively. **(B)** The LptD G580S and LptE T95I mutants carrying the mutations listed in A were constructed by phage transduction or ssDNA mutagenesis. Five-fold serial dilutions of the bacterial overnight culture were spotted onto LB plates supplemented with vancomycin or cholic acid and incubated overnight. **(C)** The LptD WT, LptD G580S, LptE WT, LptE T95I strains, and the suppressor mutants in B were subjected to Western blot analysis using an anti-LptD antibody or anti-LptE antibody. **(D)** OMV fractions were prepared from the LptD WT, LptD G580S, LptE WT, LptE T95I strains, and the suppressor mutants in B. The OMV fractions were electrophoresed in SDS-polyacrylamide gels and stained with Coomassie Brilliant Blue. **(E)** Silkworm killing activity of the LptD WT, LptD G580S, LptE WT, LptE T95I strains, and the suppressor mutants in B were examined. Silkworms (n = 10) were injected with bacterial cells (1.5 x 10^8^ CFU) and survival was monitored. The vertical axis represents the survival of silkworms and horizontal axis represents time after bacterial injection. The log-rank test p value was less than 0.05 between the LptD G580S mutant and other strains (upper graph), or between the LptE T95I mutant and other strains (lower graph).

To clarify the altered molecular functions of LptD and LptE in the LptD G580S and LptE T95I mutants, we searched for suppressor mutations against the cholic acid sensitivity of the LptD G580S and LptE T95I mutants. We isolated 47 strains resistant to cholic acid from the LptD G580S and LptE T95I mutants and determined the nucleotide sequences of *lptD* and *lptE* genes. In 10 of the 47 strains, there were amino acid substitutions in LptD and LptE, or nucleotide substitutions in the Shine-Dalgarno sequence upstream of *lptE* (**Fig 6A**). To examine whether these mutations are responsible for resistance against cholic acid, we constructed LptD G580S and LptE T95I mutants carrying these mutations by phage transduction or single-strand DNA (ssDNA) mutagenesis. The newly constructed LptD G580S and LptE T95I mutants carrying these mutations were resistant to cholic acid compared with the parental LptD G580S and LptE T95I mutants (**Fig 6B**), indicating that the amino acid substitutions in LptD and LptE or the nucleotide substitution in the Shine-Dalgarno sequence of *lptE* suppress the cholic acid sensitivity of the LptD G580S and LptE T95I mutants. Expression analysis of LptD and LptE revealed that the LptD G580S mutant carrying -6G>A *lptE* and the LptE T95I mutant carrying -9G>A *lptE* decreased the expression of both LptD and LptE (**Fig 6C**). The LptD G580S and LptE T95I mutants carrying the amino acid substitutions of LptD or LptE decreased the expression of LptD (**Fig 6C**). These results suggest that the suppressor mutations decrease the LptD expression to restore the cholic acid resistance. The suppressor mutants not only restored cholic acid resistance, but also lost vancomycin resistance (**Fig 6B**). Furthermore, these suppressor mutants decreased the production of OMVs and exhibited decreased killing activity against silkworms as compared with the parental LptD G580S and LptE T95I mutants (**Fig 6D and 6E**). These suppressor mutants showed indistinguishable growth from the parental LptD G580S and LptE T95I mutants in nutrient broth (**S4 Fig**). Therefore, the decreased expression of LptD caused by the suppressor mutations cancels the vancomycin resistance, the increased production of OMVs, and the increased killing activity against silkworms in the LptD G580S and LptE T95I mutants without affecting growth capability. Because decreased expression of LptD canceled the phenotypes of LptD G580S and LptE T95I, LptD G580S and LptE T95I are not considered to be loss-of-function mutations, but rather gain-of-function mutations.

### Amino acid substitutions of LptD and LptE present in *E*. *coli* clinical isolates affect *E*. *coli* resistance to antimicrobial substances

**Fig 7:**
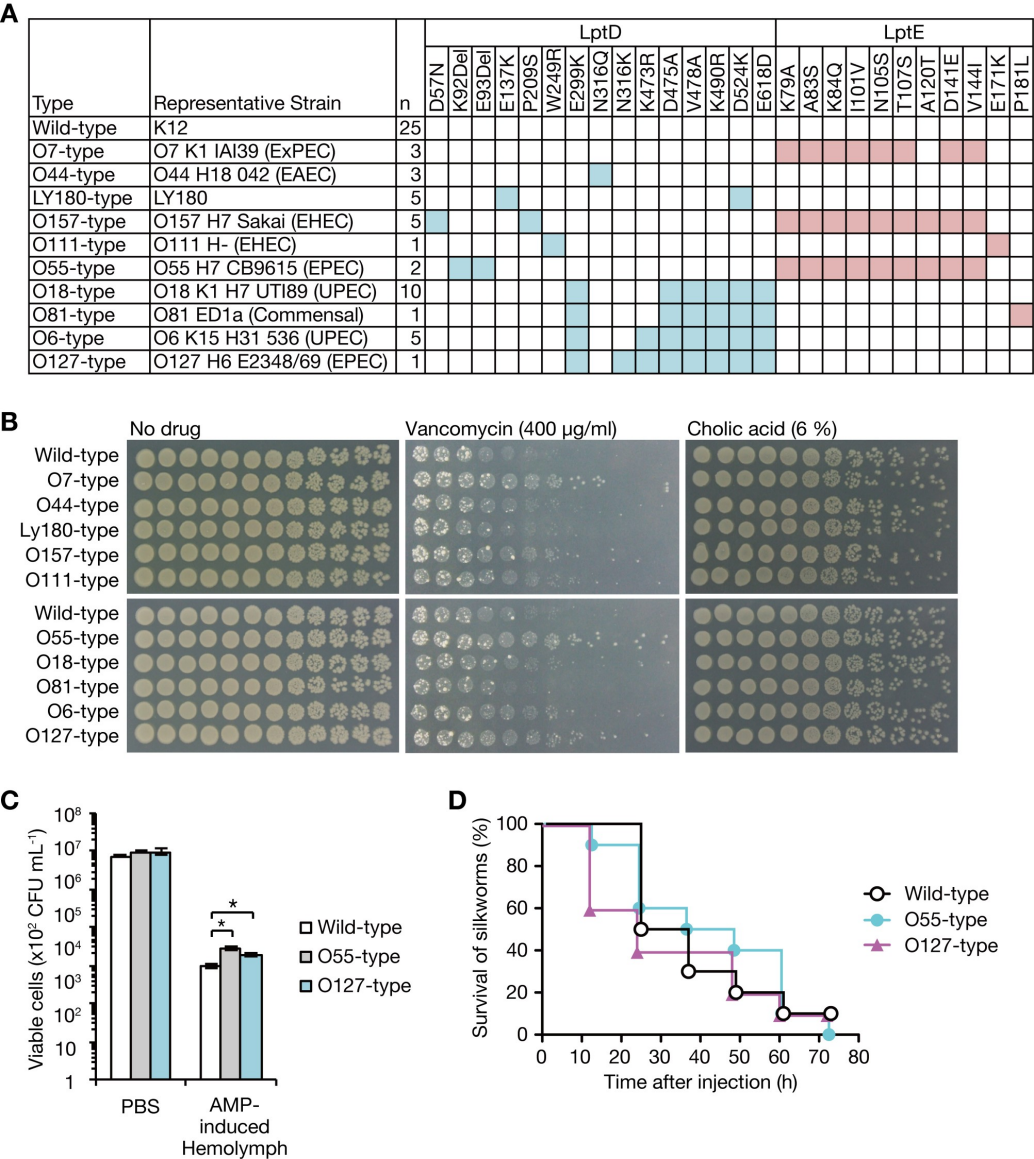
*E*. *coli* clinical isolates carry LptD and LptE mutations conferring resistance against antimicrobial substances. **(A)** Amino acid substitutions of LptD and LptE that are present in 61 *E*. *coli* strains (KEGG database) are shown. “Representative strain” is one of the strains carrying the mutations. “n” indicates the number of strains carrying identical mutations. **(B)** *E*. *coli* strains carrying the LptD and LptE mutations listed in A were constructed by ssDNA mutagenesis from a laboratory strain of *E*. *coli* (BW25113, wild-type). The bacterial strains were cultured overnight and 5-fold serial dilutions were spotted onto LB plates supplemented with vancomycin or cholic acid. **(C)** *E*. *coli* strain (BW25113) and *E*. *coli* strains carrying O55-type or O127-type mutations were incubated in 1.7% silkworm hemolymph in which AMP was induced (AMP-induced Hemolymph) or in PBS, and the number of viable bacterial cells was determined. Data shown are the mean ± standard errors from two independent experiments performed in duplicate. The asterisk represents a p value less than 0.05 (Student’s *t* test). **(D)** Silkworm killing activity of *E*. *coli* strain (BW25113) and *E*. *coli* strains carrying O55-type or O127-type mutations was examined. Silkworms (n = 10) were injected with bacterial cells (4.1 x 10^8^ CFU) and survival was monitored. The log-rank test p value was larger than 0.05 between three strains.

To explore the possibility that amino acid substitutions of LptD and LptE occur in clinical *E*. *coli* isolates, we examined the LptD and LptE amino acid sequences of *E*. *coli* strains whose genome information was available in public databases. We found several amino acid substitutions in LptD and LptE and classified the mutations into several patterns (**Fig 7A**). We constructed *E*. *coli* mutants carrying the amino acid substitutions from an *E*. *coli* laboratory strain and examined their sensitivity to vancomycin and cholic acid. The *E*. *coli* mutants carrying the O7-type, O157-type, O55-type, and O127-type mutations showed better growth than the parent strain in the presence of vancomycin (**Fig 7B**). In contrast, there was no growth difference between mutants and the parent strain in the presence of cholic acid (**Fig 7B**). The O7-type, O157-type, and O55-type mutations contained the common amino acid substitutions of LptE that are assumed to contribute to vancomycin resistance (**Fig 7A**); therefore, we used the O55-type mutant as a representative strain for further analysis. We then examined whether the O55-type and O127-type mutants are resistant to antimicrobial peptides and thus have increased virulence in a silkworm model. The number of viable cells of the O55-type and O127-type mutants was greater than that of the parent strain in the silkworm hemolymph containing antimicrobial peptides (**Fig 7C**), but the killing ability against silkworms was not increased (**Fig 7D**). These results suggest that amino acid substitutions of LptD and LptE found in clinical *E*. *coli* isolates contribute to resistance against extracellular antimicrobial substances, but the resistance level is not sufficient to confer high killing ability against silkworms.

Next, we tried to identify the substitution(s) responsible for resistance to antimicrobial substances. Because the O7-type, O157-type, and O55-type mutations contained common amino acid substitutions in LptE that are assumed to contribute to vancomycin resistance, we examined the vancomycin resistance of *lptE* mutants carrying each amino acid substitution from the common mutations. The LptE A83S/A84Q mutant exhibited better growth than the parent strain in the presence of vancomycin (**S5 Fig**), indicating that either or both A83S and A84Q are responsible for the vancomycin resistance. The O127-type mutant had slightly better growth than the O6-type mutant in the presence of vancomycin (**Fig 7B**), suggesting that LptD N316K mutation, the difference between the two types of mutations, contributes to vancomycin resistance.

### Amino acid substitutions conferring high virulence properties locate in loop 4 or the β-barrel in the LptD-LptE complex

**Fig 8:**
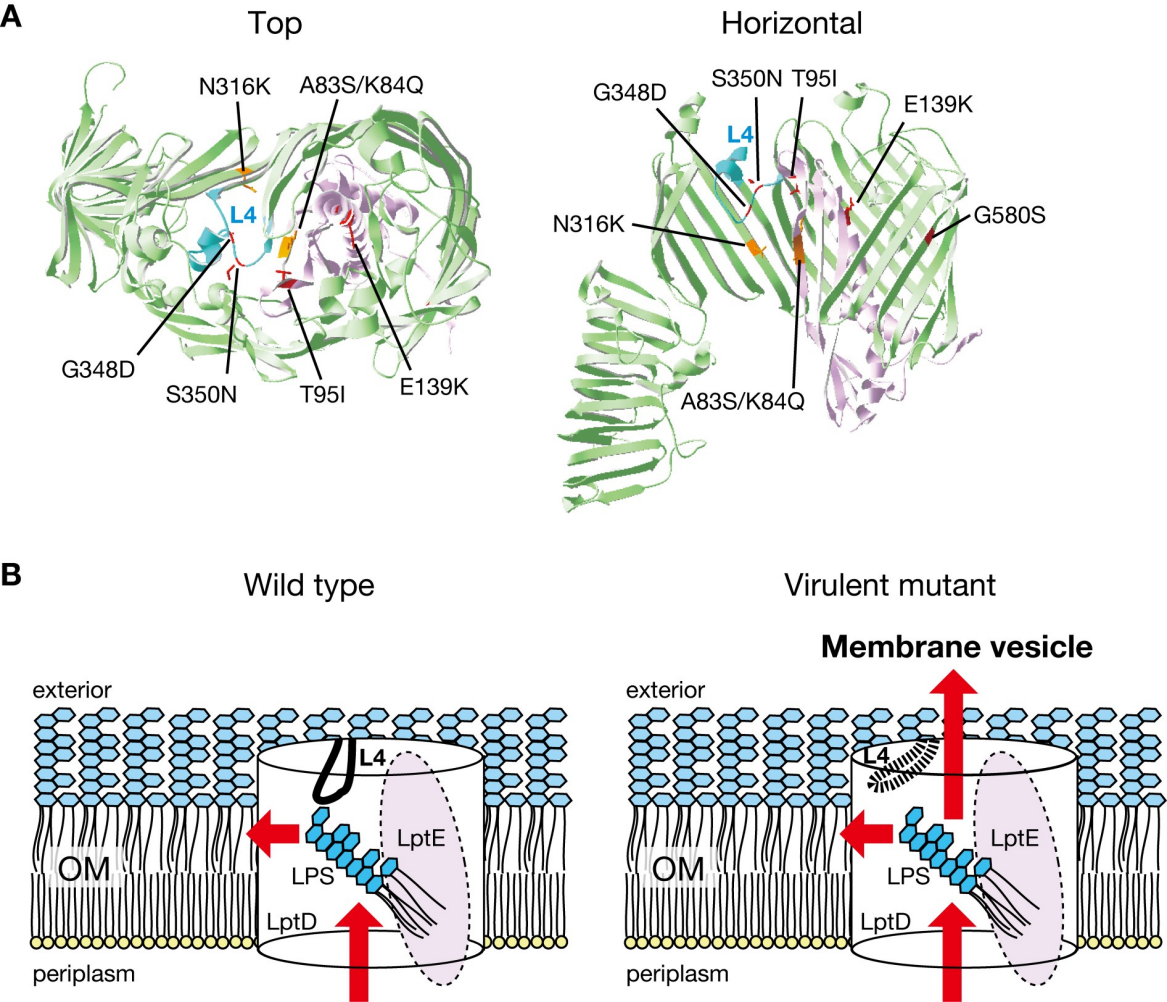
Location of the LptD and LptE mutations in the LptD-LptE complex structure. **(A)** The structural location of the LptD and LptE mutations that increase *E*. *coli* virulence is presented. The LptD-LptE complex structure is from *S*. *flexneri* (4rhb). The left image is from the exterior side, and the right image is from the horizontal side. In the right image, LptD 635–784 was removed for clarity. Loop 4 of LptD is shown in cyan, the other parts of LptD are shown in moss green, and LptE is shown in purple. The mutations obtained in the experimental evolution are in red, and the mutations present in clinical isolates are in orange. **(B)** Model for structural alteration and LPS translocation in the LptD and LptE mutants. In the wild-type strain, loop 4 plugs the LPS tunnel and assists in the translocation of LPS into the outer membrane (left). In the LptD and LptE mutants, the structure of loop 4 is changed, thereby attenuating its plug function and allowing LPS to be secreted into the extracellular milieu (right).

To gain more insights into the LptD and LptE amino acid substitutions conferring high virulence properties, we searched for other LptD and LptE mutations causing high killing activity of *E*. *coli* against silkworms by performing a single-round mutagenesis and infection experiment (**Fig 1A**). We identified that LptD G348D, LptD S350N, and LptE E139K have high killing activity in *E*. *coli* against silkworms (**S6A Fig**). These amino acid substitutions increased *E*. *coli* resistance to vancomycin (**S6B Fig**), sensitized *E*. *coli* against cholic acid (**S6B Fig**), and increased the production of OMVs (**S6C Fig**). Therefore, LptD G348D, LptD S350N, and LptE E139K increase *E*. *coli* virulence in a similar manner as LptD G580S and LptE T95I. Next, we mapped the LptD and LptE mutations in the LptD-LptE complex structure. LptD G348 and LptD S350 were present on loop 4 of LptD (**Fig 8A**). LptE T95 interacted with loop 4 of LptD [23] (**Fig 8A**). In contrast, LptD G580 was present in the β-barrel of LptD, which was distant from loop 4 (**Fig 8A**). Also, LptE E139 interacted with LptD S533 present in the β-barrel of LptD [23]. Together, the amino acid substitutions of LptD and LptE identified by experimental evolution locate around loop 4 or the β-barrel of LptD. The LptE A83/K84 and LptD N316, which were identified in *E*. *coli* clinical isolates, located in the tunnel that LPS passes through, which was distant from loop 4 of LptD (**Fig 8A**).

## Discussion

In the present study, we performed an experimental evolution of pathogenic bacteria using an animal infection model and identified mutations of the growth-essential LPS transporter as causing high virulence properties of *E*. *coli*. Furthermore, this study revealed that amino acid substitutions of the LPS transporter were present in *E*. *coli* clinical isolates and contributed to resistance against antimicrobial substance. This is the first report to demonstrate that mutations in growth-essential genes can increase the bacterial virulence potential.

We found that LptD and LptE mutants were resistant to several antibiotics, host antimicrobial substances, and an organic solvent. The LptD and LptE mutants increased OMV production and increased efflux activity of isoprenoids together with OMVs. In addition, the suppressor mutants of the LptD and LptE mutants exhibited decreased OMV production, decreased resistance against vancomycin, and attenuated killing activity against silkworms. OMVs adsorb various antimicrobial molecules at the outside of bacteria and confer bacterial resistance to antimicrobial molecules [19]. Also, OMVs function to extrude intracellular molecules to the extracellular milieu [24]. On the basis of our findings and previous reports, we assume that the increased production of OMVs in the LptD and LptE mutants causes bacterial resistance against antimicrobial molecules and results in high virulence in silkworms.

LptD G580S, LptD G348D, LptD S350N, LptE T95I, and LptE E139K induced the high production of OMVs. Among these mutations, LptD G348, LptD S350N, and LptE T95I locate around loop 4 of LptD (**Fig 8A**). In addition, in the LptD G580S mutant, the interaction between loop 4 of LptD and LptE T86 was decreased compared with that in the parent strain (**Fig 5B**). In the LptE T95I mutant, the interaction between loop 4 of LptD and LptE T86 was altered compared with that in the parent strain (**Fig 5C**). These results suggest that most of the LptD and LptE mutants isolated in this study had an altered LptD loop structure. Loop 4 of LptD acts as a plug for the LPS tunnel at the exterior side and assists the translocation of LPS to outer membrane [25]. Thus, we assume that the altered structure of loop 4 decreased the function of the plug against the LPS tunnel, and LPS was secreted into extracellular space as OMVs (**Fig 8B**).

The LptD G580S and LptE T95I mutants were resistant to several antibiotics, host antimicrobial factors, and the organic solvent n-hexane, but were sensitive to cholic acid. The LptD G348D, LptD S350N, and LptE E139K mutants were also resistant to vancomycin, but sensitive to cholic acid. The antimicrobial substances to which the LptD and LptE mutants were resistant were hydrophobic molecules with a low molecular weight (e.g., tetracycline, chloramphenicol, levofloxacin, ampicillin, and n-hexane) or cationic molecules with a high molecular weight (e.g., vancomycin, antimicrobial peptide, complement, and colistin). In contrast, the substance to which the LptD and LptE mutants were sensitive, cholic acid, is an anionic molecule with a low molecular weight. These characteristics of the molecules to which the LptD and LptE mutants were resistant or sensitive can be attributed to the nature of the OMVs; (1) OMVs comprising LPS are negatively charged and can adsorb cationic molecules, but not negatively-charged molecules; (2) Lipid A, a component of LPS, is hydrophobic and can adsorb hydrophobic molecules. Therefore, it is reasonable that the increased production of OMVs in the LptD and LptE mutants contributes to resistance against cationic or hydrophobic molecules, but does not contribute to resistance to cholic acid. Then, why are the LptD and LptE mutants sensitive to cholic acid? A possible explanation is that the altered structure of loop 4 not only causes LPS to leak into the extracellular milieu, but also permits an influx of cholic acid from the LPS tunnel. Cholic acid cannot be adsorbed by the LPS due to its negative charge and accumulates in the periplasm where it causes bacterial toxicity. With regard to the vancomycin resistance of *E*. *coli*, Stokes *et al*. reported that several LPS synthesis mutants contain truncated LPS species and are resistant to vancomycin at 15˚C [26]. Because the LptD and LptE mutants obtained in this study did not contain truncated LPS species (**Fig 3A**), however, we assume that the vancomycin resistance mechanism in the LptD and LptE mutants differs from that in the LPS synthesis mutants.

Several *E*. *coli* clinical isolates had LptD N316K and LptE A83S/K84Q mutations, which confer resistance against vancomycin and antimicrobial peptides, but do not cause sensitivity to cholic acid. The LptD N316K and LptE A83S/K84Q mutations locate in the LPS tunnel, which is distal from loop 4 (**Fig 8A**). This may imply that LptD N316K and LptE A83S/K84Q do not alter the structure of loop 4 and do not permit the influx of cholic acid. In the natural habitat, *E*. *coli* exists in the mammalian colon and is exposed to bile acids containing cholic acid. Therefore, it is assumed that *E*. *coli* clinical isolates accumulate gene mutations that confer resistance against antimicrobial molecules without increasing their sensitivity to cholic acid. On the other hand, during our evolutional experiments, *E*. *coli* strains were exposed to silkworm hemolymph that did not contain cholic acid, so the high virulence mutants would easily lose their resistance against cholic acid and develop resistance against antimicrobial peptides. In this regard, we revealed that the high virulence mutants obtained by the experimental evolution did not have increased colonization capability in mouse intestine (**S1 Fig**). It is possible that the sensitivity of these mutants to cholic acid cancels the advantage of their resistance to host antimicrobial peptides or complement. Further studies are needed to verify whether the high virulence of the mutants against silkworms also results in greater virulence in mammals.

We also found *E*. *coli* clinical isolates carrying amino acid substitutions in the subunits of the LPS transporter other than LptD and LptE. LptA, LptB, LptC, LptF, and LptG, which locate in the periplasm or inner membrane, were highly conserved among *E*. *coli* strains and contained fewer amino acid substitutions than LptD and LptE (**S7 Fig**). This finding may indicate that LptD and LptE, which locate on the outer membrane, are exposed to more variable selection pressure than the other subunits and accumulate more mutations. In relation to this idea, the amino acid sequences of LptD and LptE as well as the LptD-LptE complex structure are different among *E*. *coli*, *Shigella flexneri*, *Salmonella typhimurium*, *Pseudomonas aeruginosa*, *Yersinia pestis*, and *Klebsiella pneumoniae* [27]. Based on our finding, the difference in the LptD-LptE complex structure might contribute to the virulence properties of each pathogenic bacterium, and should be examined in future studies.

In this study, we obtained a 500-fold high virulence bacterium from a non-pathogenic bacterium by experimental evolution using an animal infection model. The method is a powerful tool for observing the process in which non-pathogenic bacterium accumulates gene mutations to increase virulence properties. Future studies to identify gene mutations that cause high virulence properties, as well as to investigate the interactions among the gene mutations will advance our understanding of how bacteria acquire virulence functions.

## Methods

### Ethics statement

This study was carried out in strict accordance with the recommendation in the Fundamental Guidelines for Proper Conduct of Animal Experiment and Related Activities in Academic Research Institutions under the jurisdiction of the Ministry of Education, Culture, Sports, Science and Technology, 2006. All mouse protocols followed the Regulations for Animal Care and Use of Okayama University and were approved by the Animal Use Committee at Okayama University (approval number: OKU-2019593).

### Bacterial strains and culturing conditions

*E*. *coli* strains were aerobically cultured in Lysogeny broth (LB) medium at 37˚C. *E*. *coli* strains carrying a miniTn10 marker or chloramphenicol-resistant gene from pKD3 were cultured in the presence of 50 μg/ml kanamycin or 25 μg/ml chloramphenicol. *E*. *coli* strains transformed with pKD46 or pEVOL-pBpF were cultured in the presence of 100 μg/ml ampicillin or 12.5 μg/ml chloramphenicol. The bacterial strains or plasmids used in this study are listed in S3 Table.

### Experimental evolution using a silkworm infection model

Overnight culture of the *E*. *coli* KP7600 strain (W3110 type-A, F^-^, lacI^q^, lacZΔM15, λ^-^, galK2, galT22, IN(rrnD-rrnE)1) [28] was inoculated into 100-fold amount of fresh LB broth containing 0.2% ethylmethane sulfonate (Wako Chemical, Osaka, Japan) and incubated overnight. The ethylmethane sulfonate-treated bacterial culture was inoculated into a 100-fold amount of fresh LB broth. The overnight culture was appropriately diluted with saline, and 0.05 ml of bacterial solution was injected into silkworms *via* an intra-hemolymph route. The silkworms were maintained at 37˚C. One of the dead silkworms was dissected and the hemolymph was spread on agar plate. The plate was incubated overnight at 37˚C, and at least 10 colonies appearing on the plate were suspended in LB broth. The bacterial suspension was used for the next round of mutagenesis and infection of silkworms. The bacterial suspension in each round of the mutagenesis and infection was streaked on LB plates to obtain single colonies, which were subjected for genetic and phenotypic analyses.

### Evaluation of killing activity of *E*. *coli* strains against silkworms

Fifth instar silkworms (Fu/Yo × Tsukuba/Ne) were raised from fertilized eggs (Ehime Sansyu, Ehime, Japan) at 27˚C in our laboratory [7, 8]. The 5^th^ instar silkworms were administered an antibiotic-free artificial diet (Sysmex Corp., Kobe, Japan) for 1 day and used for the infection experiment. *E*. *coli* strains isolated in the experimental evolution were streaked on LB agar plates to obtain a single colony. The single colony was inoculated into LB broth and cultured overnight. The bacterial culture was centrifuged at 4050 *g* for 10 min to collect bacterial cells. The bacterial cells were suspended in saline. Serially diluted bacterial solutions (0.05 ml) were injected into the hemolymph of silkworms using a 1-ml syringe equipped with 27-gauge needle. The silkworms were maintained at 37˚C and dead silkworms were counted at 48 h after the injection. On the day of the infection experiment, the bacterial solution used for infection was appropriately diluted with saline, spread on LB plates, and incubated overnight at 37˚C. Colonies were counted to calculate the number of CFU injected into silkworms. Logistic regression analysis on the dose-survival plots was performed to determine LD_50_.

### Whole genome sequencing

Genomic DNA of KP7600, HV1, HV10, and HV11 strains was extracted using the PureLink Genomic DNA Mini Kit (Invitrogen). Genomic DNA libraries of these strains were prepared using the Ion Xpress Plus Fragment Library Kit (Life Technologies). Sequencing was performed using an Ion PGM sequencer (Life Technologies). At least 100 million base sequences of 100-base single end reads were generated per sample. Genomic DNA of other mutant strains isolated after the second and further rounds of mutagenesis and infection was extracted using the QIAamp DNA Blood Mini Kit (Qiagen). The genomic DNA libraries of these mutant strains were prepared using the Nextera XT DNA Library Prep Kit (Illumina, San Diego, CA, USA) and sequenced using MiSeq (Illumina). At least 100 million base sequences of 300-base paired end reads were generated per sample. The data were analyzed using the CLC Genomics Workbench software. The reads were mapped to a reference genome of the *E*. *coli* W3110 strain and the single nucleotide polymorphism causing amino acid substitutions were identified using the basic variant detection program in the CLC software. The sequencing reads were deposited in DDBJ (KP7600, HV1, HV10, HV11 strains, DRA008387; 16 strains after the second or more round of mutagenesis, DRA005482 [round 2, SAMD00071302; round 3, SAMD00071303; round 4, SAMD00071304; round 5, SAMD00071305 and SAMD00071306; round 6, SAMD00071307; round 7, SAMD00071308-SAMD00071311; round 11, SAMD00071312 and SAMD00071313; round 16, SAMD00071314 and SAMD00071315; round 21, SAMD00071316 and SAMD00071317]).

### Analysis of *E*. *coli* resistance against antimicrobial substances

We examined *E*. *coli* resistance against silkworm antimicrobial peptides according to our previous method with minor modifications [30]. First, autoclaved *E*. *coli* cells (KP7600) were injected into silkworms. Silkworm hemolymph was collected at 1 d after the injection, supplemented with phenylthiourea (final concentration 0.5 mM), frozen in liquid nitrogen, and stored at -80˚C. *E*. *coli* overnight cultures (50 μl) were inoculated into fresh LB broth (5 ml) and aerobically cultured at 37˚C for 70 min. The logarithmically growing *E*. *coli* cells were collected by centrifugation at 21,500 *g* for 1 min at 4˚C and suspended in PBS. The bacterial suspension was inoculated into PBS or silkworm hemolymph and incubated at 37˚C for 30 min. The sample was appropriately diluted with saline, spread on LB agar plates, and incubated overnight at 37˚C. The numbers of colonies that appeared were counted.

To examine *E*. *coli* resistance against serum complement, a slightly modified protocol from our previous method was used [31]. Freshly prepared porcine serum was purchased from Nippon Bio-test Laboratories Inc (Saitama, Japan). To inactivate complement activity, the serum was incubated at 56˚C for 30 min. *E*. *coli* logarithmically growing cells were prepared by the method described above. *E*. *coli* cell suspension was inoculated into PBS, porcine serum, or heat-treated porcine serum for 45 min at 37˚C. The sample was appropriately diluted with saline, spread on LB agar plates, and incubated overnight at 37˚C. The number of colonies was counted to calculate the viable cell number in the samples.

To examine *E*. *coli* resistance against antibiotics and cholic acid, autoclaved LB agar was mixed with antibiotics or cholic acid and poured into a disposable square dish (Eiken Chemical Co., ltd., Tokyo, Japan). *E*. *coli* overnight cultures were 5-fold serially diluted with LB broth in a 96-well plate. The serially diluted bacterial solution was spotted onto the LB agar plates supplemented with antibiotic or cholic acid using a 12-channel pipette. The plates were incubated at 37˚C.

To examine *E*. *coli* resistance against n-hexane, we used a previously described method with minor modifications [18]. *E*. *coli* overnight cultures (100 μl) were inoculated into a fresh LB broth (10 ml) supplemented with MgCl_2_ (final 100 mM) and glucose (final 1%) in a glass bottle (100 ml), and cultured at 37˚C. At 1 h after the inoculation, n-hexane (10 ml) was added. The optical density values at 600 nm (OD_600_) were measured every 1 h.

### Isolation of suppressor mutants

Overnight cultures of the LptD G580S and LptE T95I mutants were inoculated into LB broth supplemented with or without ethylmethane sulfonate (final concentration 0.2%). The overnight culture was inoculated into fresh LB broth and aerobically cultured overnight. The overnight culture was spread on LB agar plates supplemented with cholic acid (final concentration 6%) and incubated overnight. A colony was streaked on LB plates to isolate single colonies. The single colony was inoculated into LB broth and cultured overnight. The culture was used as the stock of bacterial strains and extraction of genome DNA. The *lptD* and *lptE* genes were amplified by polymerase chain reaction (PCR) from the genome DNA using primers (lptD-F, lptD-R, lptE-F, and lptE-R) (**S4 Table**).

### Genetic manipulation

#### Construction of the LptD and LptE mutants by phage transduction

The miniTn10 marker located near the *lptD* gene in JD20181 (yabP::miniTn10) [32] was transferred to HV1, HV3, and HVK9a, which carry LptD mutations, by transduction with phage P1 *vir* [33]. The miniTn10 marker located near the *lptE* gene in JD26070 (cobC::miniTn10) [32] was transferred to HV10 and HVK9b, which carry LptE mutations, by transduction. Transductants carrying the LptD or LptE mutations were selected by sequencing the *lptD* and *lptE* genes. The miniTn10 in the selected transductants was transferred to the KP7600 strain by transduction. Transductants carrying the LptD or LptE mutations were selected by sequencing the *lptD* and *lptE* genes, resulting in the LptD G580S, LptD G348D, LptD S350N, LptE T95I, and LptE E139K mutants.

#### Construction of the suppressor mutants by phage transduction

The miniTn10 marker (yabP::miniTn10) in the LptD G580S mutant carrying the LptD R429H, LptD G489D, or LptD E509K mutations was transferred to KP7600 by transduction, resulting in the LptD G580S suppressor mutants carrying the LptD R429H, LptD G489D, or LptD E509K mutations. The miniTn10 marker (cobC::miniTn10) in the LptE T95I mutant carrying the *lptE* -9G>A mutation was transferred to KP7600 by transduction, resulting in the LptE T95I suppressor mutant carrying the *lptE* -9G>A mutation.

For the LptD G580S mutant carrying the LptE W21R mutation, a chloramphenicol-resistant marker was introduced to the genomic region near to the *lptE* gene by Red-recombinase mediated targeting using targeting primers (**S4 Table**) [34]. The chloramphenicol resistance marker was transferred to the LptD G580S mutant by transduction, resulting in the LptD G580S suppressor mutant carrying the LptE W21R mutation. For the LptE T95I mutants carrying the LptD G412D, LptD R429H, or LptD E509K mutations, the chloramphenicol resistance marker was introduced into the genomic region near to the *lptD* gene by Red-recombinase mediated targeting using primers (**S4 Table**). The chloramphenicol resistance marker was transferred to the LptE T95I mutant by transduction, resulting in the LptE T95I suppressor mutants carrying the LptD G412D, LptD R429H, or LptD E509K mutations.

Because the LptD G580S mutants carrying the LptD G445D or *lptE* -6G>A mutations showed resistance against phage P1 *vir*, we introduced these mutations to the LptD G580S mutant by ssDNA mutagenesis [35, 36] using oligonucleotides (**S4 Table**) (details described below), resulting the LptD G580S suppressor mutants carrying the LptD G445D or *lptE* -6G>A mutations.

#### Construction of the LptD and LptE mutants by ssDNA mutagenesis

ssDNA mutagenesis was performed according to the previous methods [35, 36]. *E*. *coli* strains were transformed with pKD46 [34] expressing Red recombinase and were aerobically cultured in 30 ml of LB broth for 2.5 h at 30˚C. L-Arabinose was added to the culture (final 0.15%). The culture was incubated for 30 min, cooled on ice for 40 min, and centrifuged at 1800 *g* for 15 min at 4˚C. The precipitated cells were suspended in ice-cold water and centrifuged at 3000 *g* for 5 min at 4˚C. This washing procedure was repeated and the cells were finally suspended in 300 μl of ice-cold water. The cell suspension (50 μl) was mixed with 10 pmol of ssDNA coding the intended amino acid substitutions and three to four synonymous codon mutations (**S4 Table**), electroporated (2.5 kV, 200 Ω, 25 μFD), and suspended in 1 ml of SOC medium. The bacterial cells were cultured at 30˚C for 30 min, and spread on LB agar plates. After incubation overnight, the colonies were suspended in water in a 96-well plate and the strain carrying the desired mutation was screened by mismatch amplification mutation assay-PCR [36]. The desired mutation was confirmed by sequencing. pKD46 was removed from the mutant cells by culturing at 37˚C.

### Evaluation of colonization ability of *E*. *coli* strains in mouse intestine

The mouse colonization experiment was performed according to the previous method [29] with minor modifications. ICR male mice (6 weeks of age) were given drinking water containing kanamycin monosulfate (0.6 mg/ml) for 24 h, and administered 0.2 ml of bacterial solution (10^7^ CFU) by gavage. The mice were individually housed in clean cages. On days 1, 3, and 6 after the bacterial administration, their feces were collected for 2 h and weighed. The feces were homogenized in 10 ml of saline, serially diluted with saline, and plated onto MacConkey agar plates containing kanamycin (100 μg/ml). Feces from mice not administered the bacterial solution did not produce colonies in this assay.

To perform competition assay, chloramphenicol resistance marker was transferred to LptD WT, LptD G580, LptE WT, LptE T95I strains by transduction (**S3 Table**). Mice were administered 0.2 ml of bacterial solution (10^7^ CFU) consisted of chloramphenicol-sensitive and -resistant strains at the ratio of 1:1. On day 1 after the bacterial administration, their feces were collected for 2 h and weighed. The feces were homogenized and plated onto MacConkey agar plated containing kanamycin (100 μg/ml) and MacConkey agar plates containing kanamycin (100 μg/ml) and chloramphenicol (12.5 μg/ml). The number of chloramphenicol-sensitive colonies was calculated by subtracting the number of colonies obtained on an agar plate without chloramphenicol by the number of colonies obtained on an agar plate with chloramphenicol. Competitive index was calculated by dividing the number of colonies resistant to chloramphenicol by the number of colonies sensitive to chloramphenicol.

### Preparation of OMVs

*E*. *coli* overnight culture (1 ml) was inoculated into 100 ml of LB broth and aerobically cultured at 37˚C for 24 h. The bacterial culture was centrifuged at 16,200 *g* for 10 min, and the supernatant was filtered with a 0.45-μm polyvinylidene difluoride (PVDF) membrane (Millex-HV, Millipore). The sample was ultracentrifuged at 235,000 *g* for 3 h (45Ti rotor, Beckman). The ultracentrifuged precipitate was suspended in HEPES-NaCl (10 mM HEPES-NaOH [pH 6.8], 146 mM NaCl). The sample was mixed with 3x sodium dodecyl sulfate (SDS) sample buffer, boiled for 5 min, and electrophoresed on a 15% SDS polyacrylamide gel. The gel was stained with Coomassie Brilliant Blue. To identify the protein, the band was excised, digested with trypsin, and subjected to matrix-assisted laser desorption ionization time-of-flight mass spectrometry.

To measure OMV diameter, the OMV fraction was filtered with 0.2-μm PVDF membranes and analyzed using a dynamic light-scattering assay (Delsa Nano C, Beckman). Further purification of the OMVs was performed according to the previously method [37]. The ultracentrifuged sample was suspended in 2 ml of 45% iodixanol and poured in the bottom of tube. An iodixanol gradient was prepared on top of the sample (2 mL, 40%; 2 mL, 35%; 3mL, 30%; 2 mL, 25%, 1 mL, 20%) and was ultracentrifuged at 160,000 *g* for 18 h at 4˚C (SW40Ti rotor, Beckman). After the ultracentrifugation, 1-ml fractions were collected from the top.

### Measurement of isoprenoids in OMVs

The *E*. *coli* strains were transformed with pAC-ZEAXipi [38] or pAC-BETAipi [39]. OMV fractions were prepared from the transformed strains as described above. Two hundred microliters of the OMV fraction was mixed with 1 ml of acetone and vortexed. The sample was centrifuged at 21,500 *g* for 5 min, and the supernatant was used for measurement of isoprenoids in OMVs. To measure the amount of isoprenoids in the extracellular fraction, isoprenoids were extracted from bacterial culture supernatants by the Bligh and Deyer method [40]. The extract was evaporated and dissolved in acetone. The sample was centrifuged at 21,500 *g* for 2 min, and the supernatant was used for measurement of isoprenoids in the extracellular fraction. To determine the amount of isoprenoids in bacterial cells, bacterial cell precipitate was mixed with 10 ml of acetone, and vortexed. The sample was centrifuged at 2300 *g* for 10 min, and the supernatant was used to measure isoprenoids in bacterial cells. The amount of isoprenoids in the samples was determined by spectrophotometry (zeaxanthin, 457 nm; β-carotene, 453 nm). Standard curves were made using purified isoprenoids (zeaxanthin, ChromaDex Inc., Irvine, CA; β-carotene, Nacalai, Kyoto, Japan).

### Preparation of polyclonal antibodies for LptD and LptE

According to previous reports [15, 41], DNA fragments encoding partial LptD (319–775 aa) or partial LptE (20–193 aa) were amplified by PCR and cloned into pET28b, resulting in pET28b-LptD and pET28b-LptE. *E*. *coli* BL21Star(DE3) was transformed with each plasmid and inoculated into 1 L of LB broth for 3 h at 37˚C. Isopropyl β-D-1-thiogalactopyranoside was added to the culture (final concentration: 1 mM) and further cultured for 5 h. The recombinant His-tagged LptD or His-tagged LptE was purified from the bacterial cells using a denaturing purification method with a Ni column (Probond resin, Invitrogen). Proteins were electrophoresed on SDS-polyacrylamide gels and stained with Coomassie Brilliant Blue. Bands corresponding to the His-tagged LptD or His-tagged LptE were excised and used to immunize rabbits. Immunization was performed five times at 2-week intervals. IgG was purified from the rabbit blood by protein G-Sepharose.

### Western blot analysis

*E*. *coli* overnight cultures (50 μl) were inoculated into 5 ml of LB broth and aerobically cultured for 3 h (logarithmic growth phase) or 25 h (stationary phase) at 37˚C. The bacterial culture was centrifuged at 21,500 *g* for 2 min, and the precipitated cells were suspended in PBS and mixed with 3x SDS sample buffer. To detect mature LptD, 3x SDS sample buffer without β-mercaptoethanol was used. The bacterial cell concentration in the sample was adjusted to OD_600_ = 15. Samples were electrophoresed in SDS-polyacrylamide gels (12.5%, LptD; 15%, LptE and LPS), and the proteins or LPS were transferred to a PVDF membrane (Immobilon-P, Millipore). The membrane was treated with a blocking buffer (20 mM Tris-HCl [pH7.6], 150 mM NaCl, 0.12% Tween20, 5% skim milk) for 1 h with gentle shaking at room temperature. The membrane was treated with a blocking buffer containing 1:1000 anti-LptD IgG, 1:3000 anti-LptE IgG, or 1:1500 anti-LPS core (WN1 222–5, Hycult Biotech) for 1 h at room temperature. After washing with TBST (20 mM Tris-HCl [pH7.6], 150 mM NaCl, 0.12% Tween20), the membrane was treated with a blocking buffer containing 1:7000 anti-rabbit IgG conjugated with alkaline phosphatase (Promega) or 1:5000 anti-mouse IgG conjugated with alkaline phosphatase for 30 min at room temperature. After washing with TBST, the membrane was treated with a staining buffer (100 mM Tris-HCl [pH9.5], 100 mM NaCl, 50 mM MgCl2, 2% nitro-blue tetrazolium/5-bromo-4-chloro-39-indolyphosphate).

### *In vivo* cross-linking analysis

*In vivo* cross-linking experiments were performed according to the previously described method with minor modifications [22]. The LptD WT, LptD G580S, LptE WT, and LptE T95I strains were transformed with pKD46 and pEVOL-pBpF. The transformed strains were cultured in LB broth containing 10 mM *p*BPA, and the *lptE* amber mutations were introduced into the genome by ssDNA mutagenesis as described above. Successful introduction of the intended mutations was confirmed by DNA sequencing. Overnight cultures of the *lptE* amber mutants were inoculated into LB broth containing 10 mM *p*BPA and 0.02% L-arabinose, and cultured for 24 h. The culture was centrifuged at 21,500 *g* for 2 min and the cells were suspended in Tris-buffered saline (20 mM Tris-HCl [pH7.6], 150 mM NaCl). Half of the cell suspension was irradiated with UV light for 10 min (B-100AP, AnalytikJena). The samples were mixed with 3x SDS sample buffer, boiled for 5 min, and electrophoresed in an SDS-polyacrylamide gel. The gel was subjected to Western blot analysis using anti-LptD antibody or anti-LptE antibody. The band intensity was measured by densitometry scanning (Image J, NIH).

## Supporting information

S1 Fig Colonization ability of the LptD and LptE mutants in mouse intestine. (A) ICR mice (n = 6) were orally administered the LptD WT, LptD G580S, LptE WT, or LptE T95I strains. The number of bacterial colonies recovered from the mouse feces was counted on days 1, 3, and 6 after the bacterial administration. Dotted lines indicate the detection limit in the assay (20 CFU/g feces). ND, not detected. (B) The LptD WT, LptD G580S, LptE WT, or LptE T95I strains were labeled with a cassette conferring resistance to chloramphenicol. The chloramphenicol-resistant strains were mixed with the chloramphenicol-sensitive LptD WT or LptE WT strains at the ratio of 1:1 and were administered to ICR mice (n = 6–7). The number of bacterial colonies recovered from the mouse feces was counted on day 1 after the bacterial administration. The competitive index was calculated by dividing the number of chloramphenicol-resistant colonies by the number of chloramphenicol-sensitive colonies. Gray circle represents the value was more than 10 or less than 0.1, because either of the chloramphenicol-resistant or -sensitive colony was not detected.
(TIF)

S2 Fig Diameter measurement of OMVs from the LptD and LptE mutants. OMV fractions of the LptD WT, LptD G580S, LptE WT, and LptE T95I strains were subjected to dynamic light-scattering analysis. The horizontal axis represents the particle diameter, and the vertical axis represents the relative distribution of the particles to the total particles.
(TIF)

S3 Fig *In vivo* cross-linking analysis of the LptD-LptE complex using an anti-LptD antibody. The LptD WT, LptD G580S, LptE WT, and LptE T95I strains expressing *p*BPA-substituted LptE were irradiated with UV light or not irradiated, and subjected to Western blot analysis using an anti-LptD antibody.
(TIF)

S4 Fig Growth curves of the LptD and LptE suppressor mutants. *E*. *coli* strains of LptD WT, LptD G580S, LptE WT, LptE T95I, and the suppressor mutants were aerobically cultured in LB broth at 37˚C. The vertical axis represents the OD_600_ of bacterial culture, and the horizontal axis represents the culture time. The growth curves of LptD WT or LptE WT are identical in this figure.
(TIF)

S5 Fig Identification of LptE mutations conferring vancomycin resistance from O55-type mutations. *E*. *coli* strains carrying O55-type mutations were constructed by ssDNA mutagenesis. The strains were cultured overnight and 5-fold serial dilutions were spotted onto LB plates supplemented with vancomycin. The left panel indicates the amino acid substitutions carried by the mutants.
(TIF)

S6 Fig Phenotypic characterization of the LptD G348D, LptD S350N, LptE E139K mutants. (A) The LptD WT, LptD G348D, LptD S350N, LptE WT, and LptE E139K strains were cultured overnight and serial dilutions of bacterial cells were then injected into silkworms. Silkworm survival was counted at 48 h after the injection. The LD_50_ value was determined by logistic regression from the dose-survival plot. Data shown are the mean ± standard errors from three independent experiments. The asterisk represents a p value less than 0.05 (Student’s *t* test). (B) Parent and mutant strains of LptD and LptE were cultured overnight and 5-fold serial dilutions were spotted onto LB plates supplemented with vancomycin or cholic acid. (C) OMV fractions of the parent and mutant strains of LptD and LptE were electrophoresed in SDS-polyacrylamide gels and stained with Coomassie Brilliant Blue.
(TIF)

S7 Fig Number of amino acid substitutions in the LPS transporter subunits in various *E*. *coli* strains. Genome data of 65 *E*. *coli* strains (KEGG database) were examined to count the number of amino acid substitutions in LptA, LptB, LptC, LptD, LptE, LptF, and LptG. Horizontal axis represents the number of amino acid substitutions, and the vertical axis represents the number of strains.
(TIF)

S1 Table Amino acid substitutions identified in high virulence mutants. (DOCX)

S2 Table Identification of proteins increased in the LptD and LptE mutants. The protein band stained with Coomassie Brilliant Blue was excised and digested in-gel with trypsin. The sample was subjected to Matrix-assisted laser desorption ionization time-of-flight mass spectrometry analysis (Microflex LRF 20, Bruker Daltonics). Database searching was performed using the Mascot search program (www.matrixscience.com).
(DOCX)

S3 Table List of bacterial strains and plasmids used. (DOCX)

S4 Table Primers used in this study. (DOCX)
