# Supplementary figures and images for: Non-pathogenic Escherichia coli acquires virulence by mutating a growth-essential LPS transporter
Source: PLoS Pathog. 2020 Apr 23;16(4):e1008469. doi: 10.1371/journal.ppat.1008469 (PMC7179839; doi:10.1371/journal.ppat.1008469)

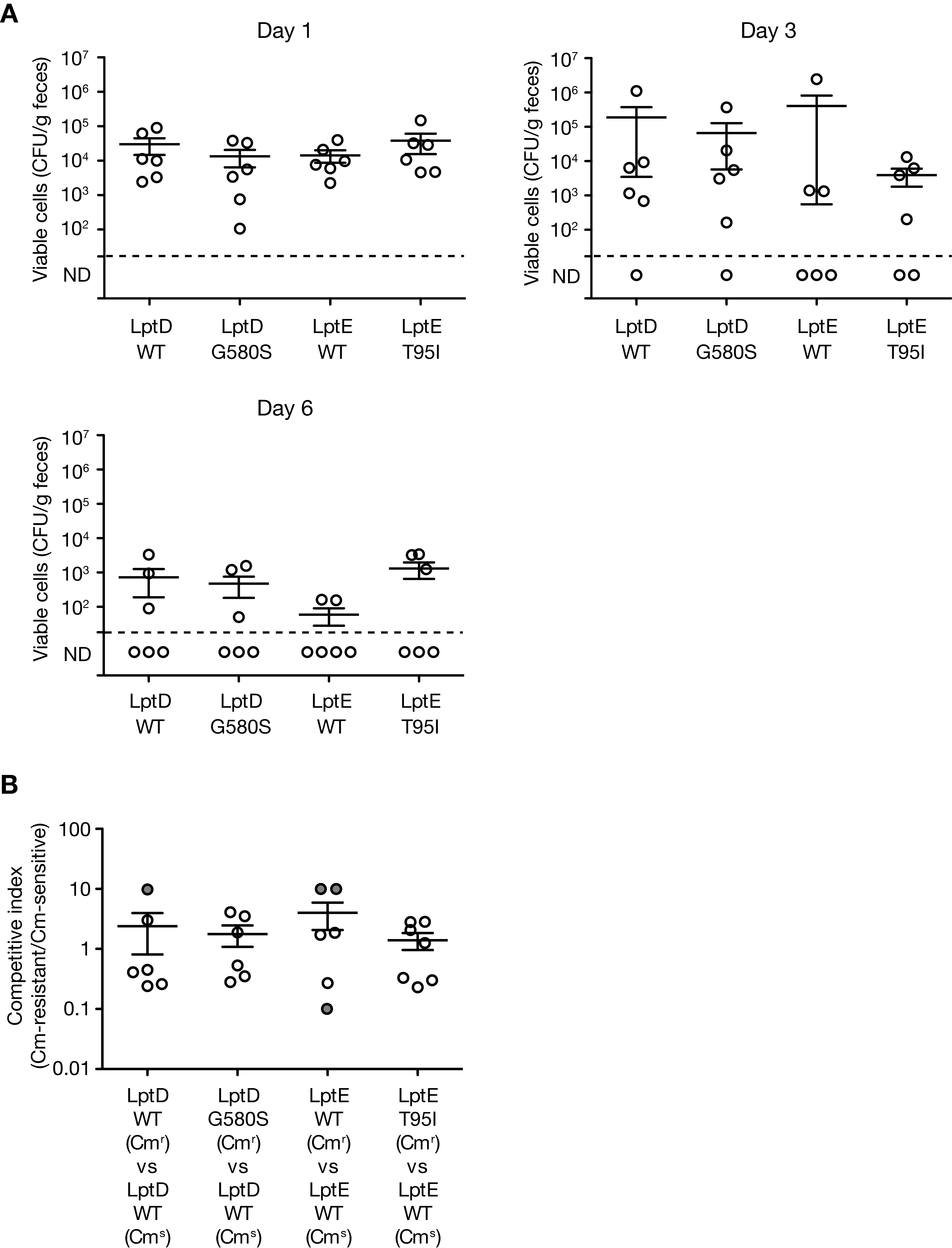

Supplement: S1 Fig — (A) ICR mice (n = 6) were orally administered the LptD WT, LptD G580S, LptE WT, or LptE T95I strains. The number of bacterial colonies recovered from the mouse feces was counted on days 1, 3, and 6 after the bacterial administration. Dotted lines indicate the detection limit in the assay (20 CFU/g feces). ND, not detected. (B) The LptD WT, LptD G580S, LptE WT, or LptE T95I strains were labeled with a cassette conferring resistance to chloramphenicol. The chloramphenicol-resistant strains were mixed with the chloramphenicol-sensitive LptD WT or LptE WT strains at the ratio of 1:1 and were administered to ICR mice (n = 6–7). The number of bacterial colonies recovered from the mouse feces was counted on day 1 after the bacterial administration. The competitive index was calculated by dividing the number of chloramphenicol-resistant colonies by the number of chloramphenicol-sensitive colonies. Gray circle represents the value was more than 10 or less than 0.1, because either of the chloramphenicol-resistant or -sensitive colony was not detected. (TIF) [file ppat.1008469.s001.tif]

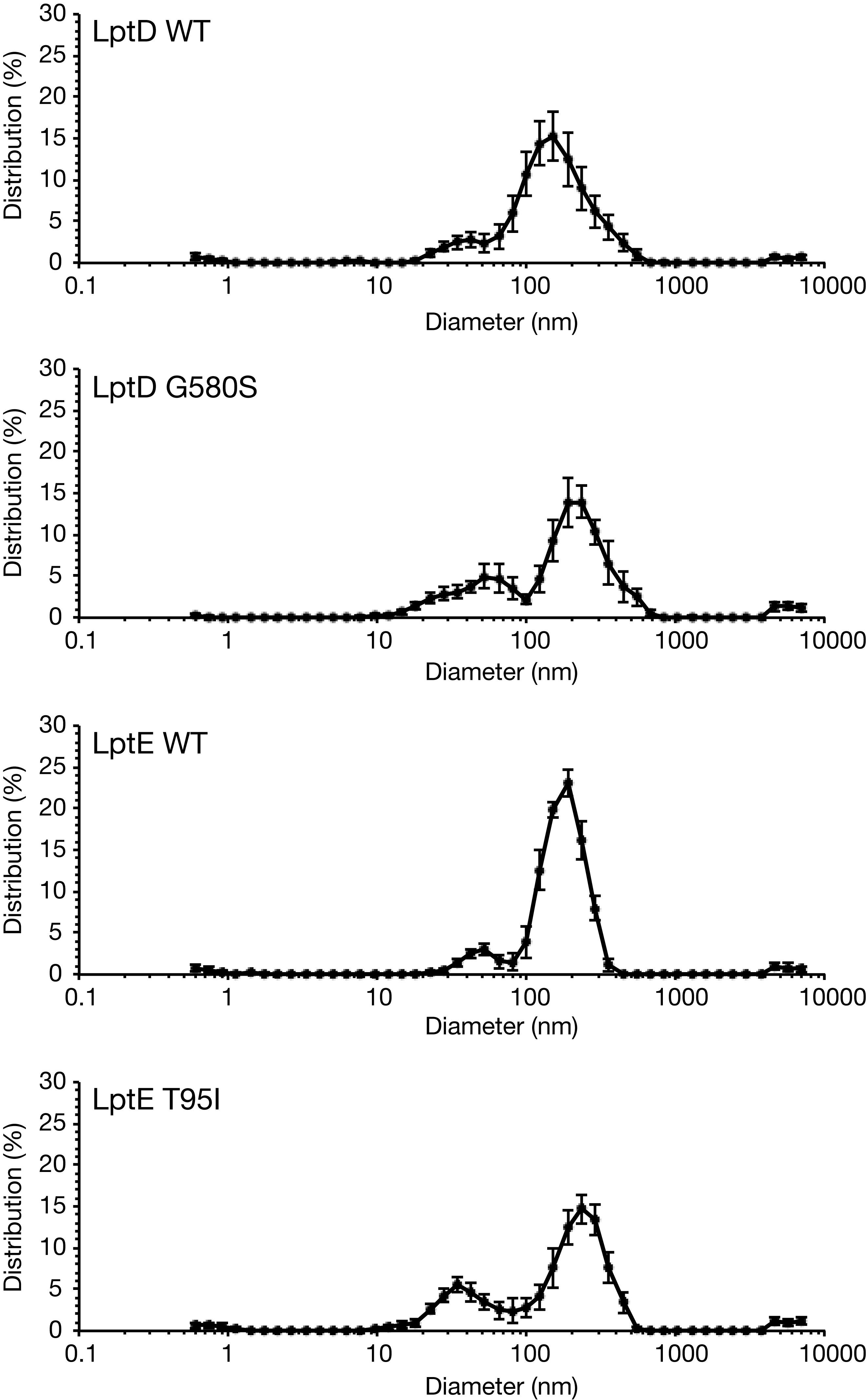

Supplement: S2 Fig — OMV fractions of the LptD WT, LptD G580S, LptE WT, and LptE T95I strains were subjected to dynamic light-scattering analysis. The horizontal axis represents the particle diameter, and the vertical axis represents the relative distribution of the particles to the total particles. (TIF) [file ppat.1008469.s002.tif]

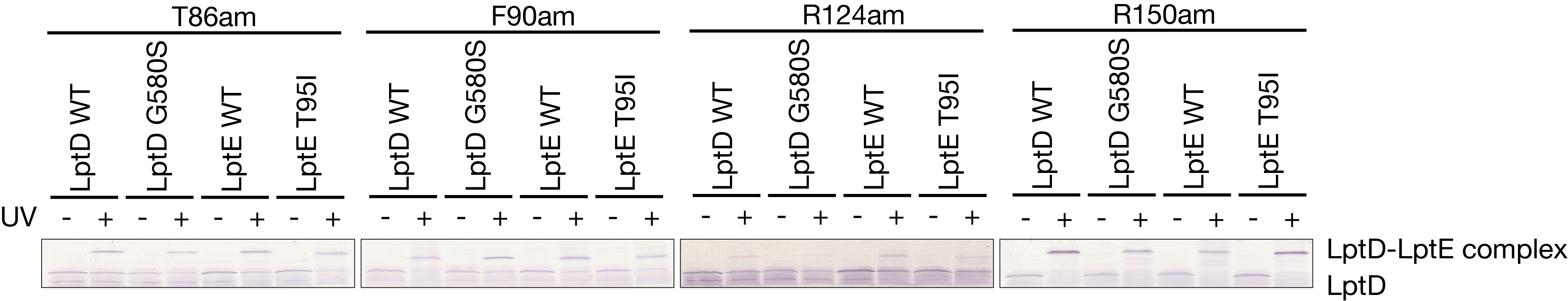

Supplement: S3 Fig — The LptD WT, LptD G580S, LptE WT, and LptE T95I strains expressing pBPA-substituted LptE were irradiated with UV light or not irradiated, and subjected to Western blot analysis using an anti-LptD antibody. (TIF) [file ppat.1008469.s003.tif]

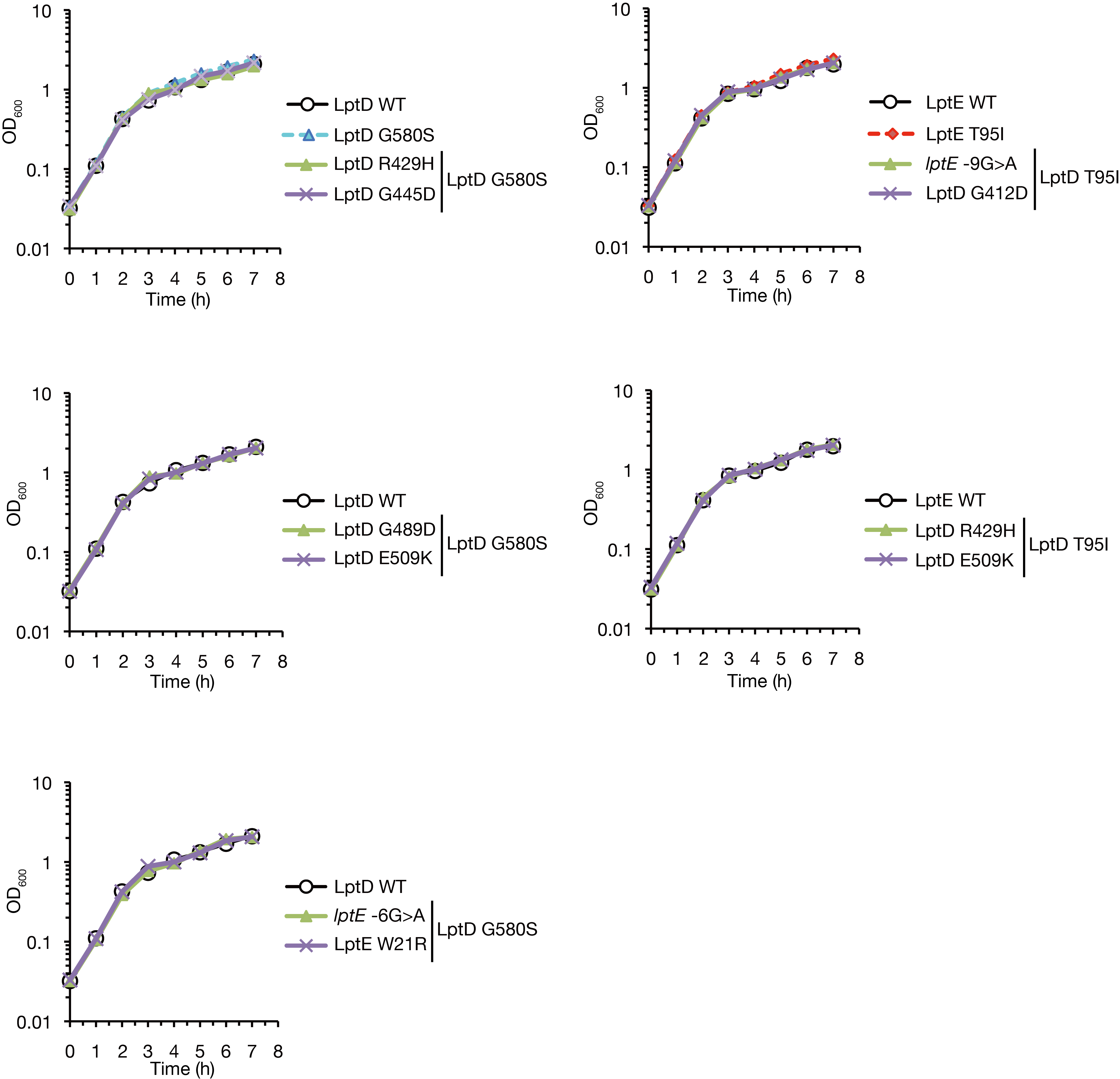

Supplement: S4 Fig — E. coli strains of LptD WT, LptD G580S, LptE WT, LptE T95I, and the suppressor mutants were aerobically cultured in LB broth at 37˚C. The vertical axis represents the OD600 of bacterial culture, and the horizontal axis represents the culture time. The growth curves of LptD WT or LptE WT are identical in this figure. (TIF) [file ppat.1008469.s004.tif]

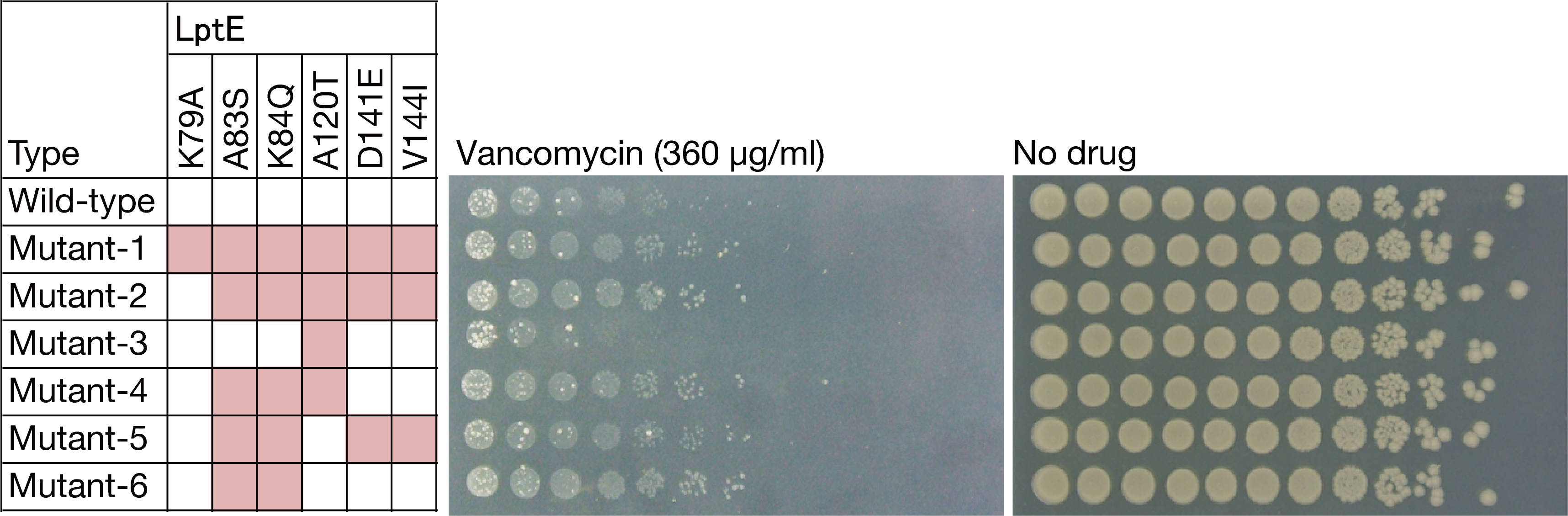

Supplement: S5 Fig — E. coli strains carrying O55-type mutations were constructed by ssDNA mutagenesis. The strains were cultured overnight and 5-fold serial dilutions were spotted onto LB plates supplemented with vancomycin. The left panel indicates the amino acid substitutions carried by the mutants. (TIF) [file ppat.1008469.s005.tif]

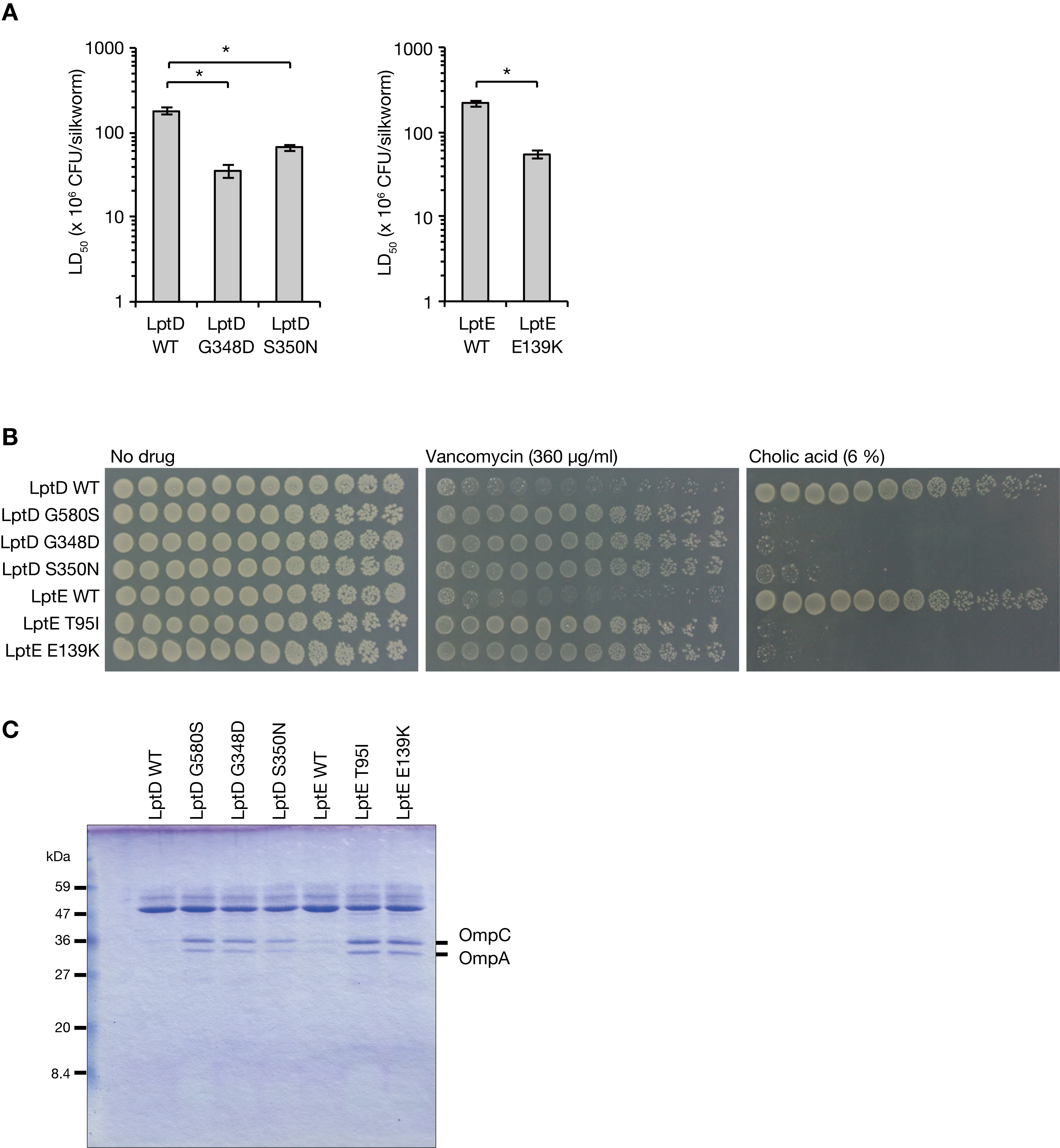

Supplement: S6 Fig — (A) The LptD WT, LptD G348D, LptD S350N, LptE WT, and LptE E139K strains were cultured overnight and serial dilutions of bacterial cells were then injected into silkworms. Silkworm survival was counted at 48 h after the injection. The LD50 value was determined by logistic regression from the dose-survival plot. Data shown are the mean ± standard errors from three independent experiments. The asterisk represents a p value less than 0.05 (Student’s t test). (B) Parent and mutant strains of LptD and LptE were cultured overnight and 5-fold serial dilutions were spotted onto LB plates supplemented with vancomycin or cholic acid. (C) OMV fractions of the parent and mutant strains of LptD and LptE were electrophoresed in SDS-polyacrylamide gels and stained with Coomassie Brilliant Blue. (TIF) [file ppat.1008469.s006.tif]

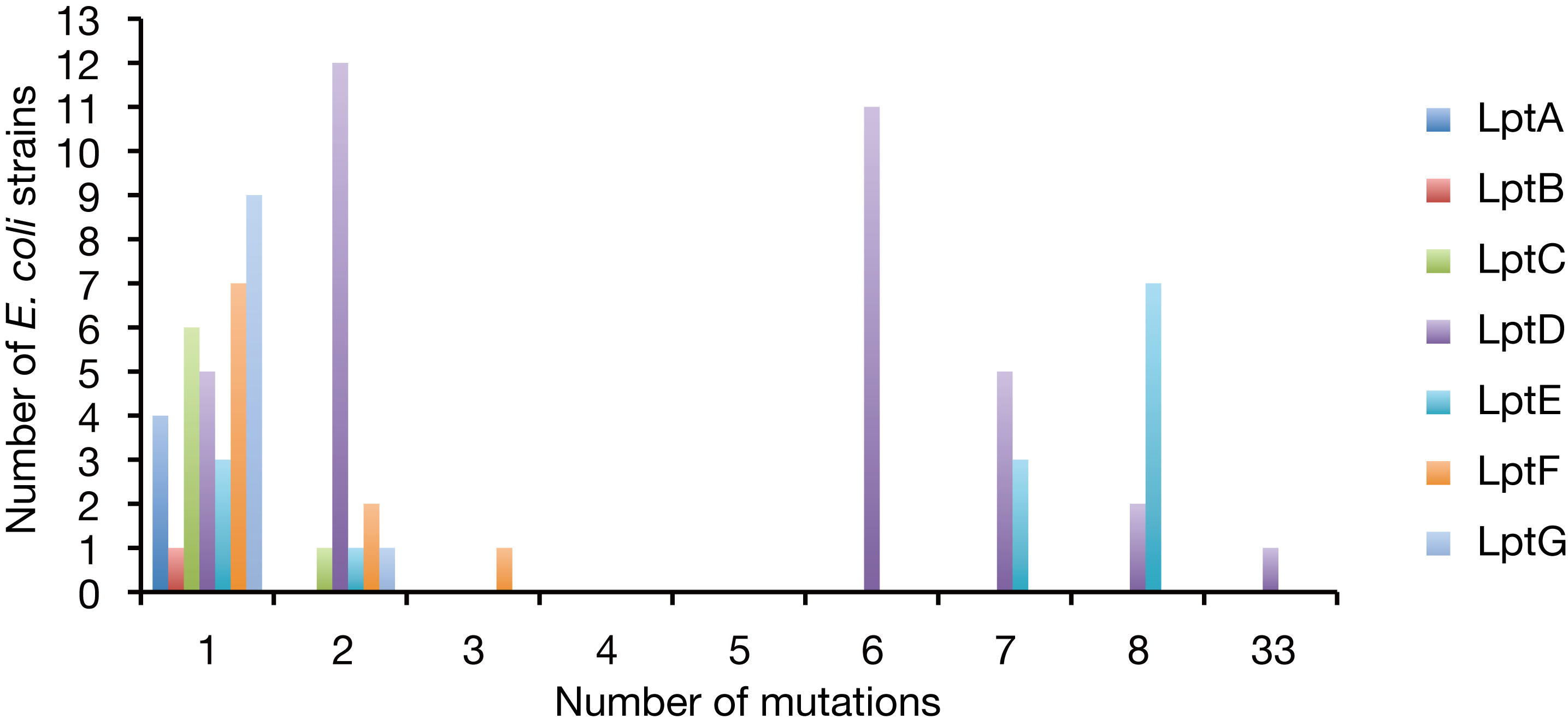

Supplement: S7 Fig — Genome data of 65 E. coli strains (KEGG database) were examined to count the number of amino acid substitutions in LptA, LptB, LptC, LptD, LptE, LptF, and LptG. Horizontal axis represents the number of amino acid substitutions, and the vertical axis represents the number of strains. (TIF) [file ppat.1008469.s007.tif]
