# Supplementary material for: Non-pathogenic Escherichia coli acquires virulence by mutating a growth-essential LPS transporter
Source: PLoS Pathog. 2020 Apr 23;16(4):e1008469. doi: 10.1371/journal.ppat.1008469 (PMC7179839; doi:10.1371/journal.ppat.1008469)
Supplement: S1 Table — (DOCX) [file ppat.1008469.s008.docx]

**S1 Table. Amino acid substitutions identified in high virulence mutants**

| Strain | Reference | SNP | Amino acid substitution | Gene and product |
| --- | --- | --- | --- | --- |
| HV1 | 55372 | C>T | G580S | lptD, exported protein required for envelope biosynthesis and integrity |
|  | 304209 | C>T | R74H | yagV, hypothetical protein |
|  | 488205 | G>A | V816M | kefA, fused mechanosensitive channel proteins |
|  | 1916014 | C>T | D173N | prc, carboxy-terminal protease for penicillin-binding protein 3 |
| HV10 | 628162 | C>T | A130V | entA, 2,3-dihydro-2,3-dihydroxybenzoate dehydrogenase |
|  | 672325 | G>A | T95I | lptE, minor lipoprotein |
|  | 1705201 | C>T | M274I | ydgJ, predicted oxidoreductase |
|  | 1784384 | G>A | A426T | ydiS, predicted oxidoreductase, FAD/NAD(P)-binding domain |
|  | 1880237 | C>T | S270L | yeaP, predicted diguanylate cyclase |
|  | 1938991 | C>T | T209I | yebK, predicted DNA-binding transcriptional regulator |
|  | 1976099 | C>T | A314T | cheA, fused chemotactic sensory histidine kinase |
|  | 2017518 | C>T | Q168Stop | fliG, flagellar motor switching and energizing component |
|  | 2846216 | G>A | A561V | hycC, hydrogenase 3, membrane subunit |
|  | 3647237 | G>A | A129T | hemD, uroporphyrinogen III synthase |
|  | 4234763 | G>A | A274T | yjbC, 23S rRNA pseudouridine synthase |
|  | 4243042 | G>A | D607N | yjbH, predicted porin |
|  | 4361245 | G>A | A684V | cadA, lysine decarboxylase 1 |
|  | 4485435 | C>T | G342G | yjgN, conserved inner membrane protein |
|  | 4519790 | G>A | A523V | fecA, ferric citrate outer membrane transporter |
| HV11 | 134958 | G>A | Q209Stop | speD, S-adenosylmethionine decarboxylase |
|  | 237849 | C>T | S172F | yafT, predicted aminopeptidase |
|  | 672325 | G>A | T95I | lptE, minor lipoprotein |
|  | 886635 | G>A | A376V | ybjJ, predicted transporter |
|  | 1087662 | C>T | G203S | ycdQ, predicted glycosyl transferase |
|  | 1579155 | C>T | G60E | yddB, predicted porin protein |
|  | 2748857 | C>T | V170I | grpE, heat shock protein |
|  | 2785512 | G>A | A37T | ygaR, hypothetical protein |
|  | 2832389 | G>A | A420T | norV, flavorubredoxin oxidoreductase |
|  | 3121936 | C>T | E176K | glcB, malate synthase G |
|  | 3526504 | C>T | R83H | cdh, CDP-diacylglycerol phosphotidylhydrolase |
|  | 3591713 | C>T | R257H | yihF, hypothetical protein |
|  | 3704036 | G>A | R444C | trkD, potassium transporter |
|  | 3711196 | C>T | A60V | gidA, glucose-inhibited cell-division protein |
|  | 4588434 | G>A | T1021M | hsdR, endonuclease R |

‘Reference’ means a position in *E. coli* W3110 genome. ‘SNP’ is single nucleotide polymorphism.
