# Supplementary material for: Non-pathogenic Escherichia coli acquires virulence by mutating a growth-essential LPS transporter
Source: PLoS Pathog. 2020 Apr 23;16(4):e1008469. doi: 10.1371/journal.ppat.1008469 (PMC7179839; doi:10.1371/journal.ppat.1008469)
Supplement: S2 Table — The protein band stained with Coomassie Brilliant Blue was excised and digested in-gel with trypsin. The sample was subjected to Matrix-assisted laser desorption ionization time-of-flight mass spectrometry analysis (Microflex LRF 20, Bruker Daltonics). Database searching was performed using the Mascot search program (www.matrixscience.com). (DOCX) [file ppat.1008469.s009.docx]

**S2 Table. Identification of proteins increased in the LptD and LptE mutants**

| Molecular weight (kDa) | | Protein predicted | Peptides mached | Sequence coverage |
| --- | --- | --- | --- | --- |
| Observed | Theoretical |  |  | (%) |
| 32.5 | 29.5 | Outer membrane protein OmpC  (gi: 545157288) | 9 | 63 |
| 30.5 | 29.5 | Outer-membrane protein OmpA  (gi: 485728270) | 13 | 56 |
| 15.0 | 16.3 | Outer-membrane protein OmpX  (gi: 57651032) | 10 | 70 |
