# Supplementary material for: Non-pathogenic Escherichia coli acquires virulence by mutating a growth-essential LPS transporter
Source: PLoS Pathog. 2020 Apr 23;16(4):e1008469. doi: 10.1371/journal.ppat.1008469 (PMC7179839; doi:10.1371/journal.ppat.1008469)
Supplement: S3 Table — (DOCX) [file ppat.1008469.s010.docx]

**S3 Table. List of bacterial strains and plasmids used**

| Strain or plasmid | Genotypes or characteristics | Source or reference |
| --- | --- | --- |
| Strains |  |  |
| KP7600 | W3110 type-A, F^-^, lacI^q^, lacZ∆M15, λ^-^, galK2, galT22, IN(rrnD-rrnE)1 | NBRP |
| HV1 | A high virulent mutant from KP7600 carrying LptD G580S | This study |
| HV10 | A high virulent mutant from KP7600 carrying LptE T95I | This study |
| HV11 | A high virulent mutant from KP7600 carrying LptE T95I | This study |
| HV3 | A high virulent mutant from KP7600 carrying LptD G348D | This study |
| HVK9a | A high virulent mutant from KP7600 carrying LptD S350N | This study |
| HVK9b | A high virulent mutant from KP7600 carrying LptE E139K | This study |
| JD20181 | KP7600 yabP::miniTn10; Kan^r^, LptD WT | NBRP |
| PHVDG580S | KP7600 yabP::miniTn10; Kan^r^, LptD G580S | This study |
| PHVDG348D | KP7600 yabP::miniTn10; Kan^r^, LptD G348D | This study |
| PHVDS350N | KP7600 yabP::miniTn10; Kan^r^, LptD S350N | This study |
| JD26070 | KP7600 cobC::miniTn10; Kan^r^, LptE WT | NBRP |
| PHVET95I | KP7600 cobC::miniTn10; Kan^r^, LptE T95I | This study |
| PHVE139K | KP7600 cobC::miniTn10; Kan^r^, LptE E139K | This study |
| JD20181R | KP7600 intergenic(insH1-Int)::cat; Cm^r^, yabP::miniTn10; Kan^r^, LptD WT | This study |
| PHVDG580SR | KP7600 intergenic(insH1-Int):cat; Cm^r^, yabP::miniTn10; Kan^r^, LptD G580S | This study |
| JD26070R | KP7600 intergenic(caiE-F)::cat; Cm^r^, cobC::miniTn10; Kan^r^, LptE WT | This study |
| PHVET95IR | KP7600 intergenic(caiE-F)::cat; Cm^r^, cobC::miniTn10; Kan^r^, LptE T95I | This study |
| AM1 | KP7600 yabP::miniTn10; Kan^r^, LptE T86am | This study |
| AM2 | KP7600 yabP::miniTn10; Kan^r^, LptE T86am, LptD G580S | This study |
| AM3 | KP7600 cobC::miniTn10; Kan^r^, LptE T86am | This study |
| AM4 | KP7600 cobC::miniTn10; Kan^r^, LptE T86am T95I | This study |
| AM5 | KP7600 yabP::miniTn10; Kan^r^, LptE F90am, | This study |
| AM6 | KP7600 yabP::miniTn10; Kan^r^, LptE F90am, LptD G580S | This study |
| AM7 | KP7600 cobC::miniTn10; Kan^r^, LptE F90am | This study |
| AM8 | KP7600 cobC::miniTn10; Kan^r^, LptE F90am T95I | This study |
| AM9 | KP7600 yabP::miniTn10; Kan^r^, LptE R124am | This study |
| AM10 | KP7600 yabP::miniTn10; Kan^r^, LptE R124am, LptD G580S | This study |
| AM11 | KP7600 cobC::miniTn10; Kan^r^, LptE R124am | This study |
| AM12 | KP7600 cobC::miniTn10; Kan^r^, LptE R124am T95I | This study |
| AM13 | KP7600 yabP::miniTn10; Kan^r^, LptE R150am | This study |
| AM14 | KP7600 yabP::miniTn10; Kan^r^, LptE R150am, LptD G580S | This study |
| AM15 | KP7600 cobC::miniTn10; Kan^r^, LptE R150am | This study |
| AM16 | KP7600 cobC::miniTn10; Kan^r^ LptE R150am T95I | This study |
| SP1 | KP7600 yabP::miniTn10; Kan^r^, LptD G580S, R429H | This study |
| SP2 | KP7600 yabP::miniTn10; Kan^r^, LptD G580S, G445D | This study |
| SP3 | KP7600 yabP::miniTn10; Kan^r^, LptD G580S, G489D | This study |
| SP4 | KP7600 yabP::miniTn10; Kan^r^, LptD G580S, E509K | This study |
| SP5 | KP7600 yabP::miniTn10; Kan^r^, LptD G580S, lptE -6G>A | This study |
| SP6 | KP7600 intergenic(insH1-Int):cat; Cm^r^, yabP::miniTn10; Kan^r^, LptD G580S, LptE W21R | This study |
| SP7 | KP7600 cobC::miniTn10; Kan^r^, LptE T95I, -9G>A | This study |
| SP8 | KP7600 intergenic(caiE-F)::cat; Cm^r^, cobC::miniTn10; Kan^r^, LptE T95I, LptD G412D | This study |
| SP9 | KP7600 intergenic(caiE-F)::cat; Cm^r^, cobC::miniTn10; Kan^r^, LptE T95I, LptD R429H | This study |
| SP10 | KP7600 intergenic(caiE-F)::cat; Cm^r^, cobC::miniTn10; Kan^r^, LptE T95I, LptD E509K | This study |
| BW25113 | rrnB, DElacZ4787, HsdR514, DE(araBAD)567, DE(rhaBAD)568, rph-1 | NBRP |
| CL1 | BW25113 O7-type (LptE K79A, A83S, K84Q, I101V, N105S, T107S, D141E, V144I) | This study |
| CL2 | BW25113 O44-type (LptD N316Q) | This study |
| CL3 | BW25113 LY180-type (LptD E137K, D524K) | This study |
| CL4 | BW25113 O157-type (LptD D57N, P209S, LptE K79A, A83S, K84Q, I101V, N105S, T107S, A120T, D141E, V144I) | This study |
| CL5 | BW25113 O111-type (LptD W249R, LptE E171K) | This study |
| CL6 | BW25113 O55-type (LptD K92Del, E93Del, LptE K79A, A83S, K84Q, I101V, N105S, T107S, A120T, D141E, V144I) | This study |
| CL7 | BW25113 O18-type (LptD E299K, D475A, V478A, K490R, D524K, E618D) | This study |
| CL8 | BW25113 O81-type (LptD E299K, D475A, V478A, K490R, D524K, E618D, LptE P181L) | This study |
| CL9 | BW25113 O6-type (LptD E299K, K473R, D475A, V478A, K490R, D524K, E618D) | This study |
| CL10 | BW25113 O127-type (LptD E299K, N316K, K473R, D475A, V478A, K490R, D524K, E618D) | This study |
| BL21Star(DE3) | F^-^, ompT, hsdSB (rB^-^, mB^-^), gal, dcm, rne131, (DE3) | Thermo Fisher |
|  |  |  |
| Plasmids |  |  |
| pAC-ZEAXipi | A vector to produce zeaxanthin, Cm^r^ | Addgene [[1](#_ENREF_1)] |
| pAC-BETAipi | A vector to produce beta-carotene, Cm^r^ | Addgene [[2](#_ENREF_2)] |
| pET28b | *E. coli* vector for protein overproduction, Kan^r^ | Novagen |
| pET28b-LptD | pET28b with partial LptD (319-775 aa) | This study |
| pET28b-LptE | pET28b with partial LptE (20-193 aa) | This study |
| pEVOL-pBpF | tRNA synthetase/tRNA pair for the *in vivo* incorporation of a photocrosslinker, p-benzoyl-l-phenylalanine, Cm^r^ | Addgene [[3](#_ENREF_3)] |
| pKD46 | λRed recombinase expression, temperature-sensitive, Amp^r^ | [[4](#_ENREF_4)] |
| pKD4 | flp-kan-flp; Kan^r^, Amp^r^ | [[4](#_ENREF_4)] |
| pKD3 | flp-cat-flp; Cm^r^, Amp^r^ | [[4](#_ENREF_4)] |

Kan: kanamycin, Cm: chloramphenicol, Amp: ampicillin.

1. Cunningham FX, Jr., Gantt E. A portfolio of plasmids for identification and analysis of carotenoid pathway enzymes: *Adonis aestivalis* as a case study. Photosynth Res. 2007;92(2):245-59. Epub 2007/07/20. doi: 10.1007/s11120-007-9210-0. PubMed PMID: 17634749.

2. Cunningham FX, Jr., Gantt E. A study in scarlet: enzymes of ketocarotenoid biosynthesis in the flowers of *Adonis aestivalis*. Plant J. 2005;41(3):478-92. Epub 2005/01/22. doi: 10.1111/j.1365-313X.2004.02309.x. PubMed PMID: 15659105.

3. Chin JW, Martin AB, King DS, Wang L, Schultz PG. Addition of a photocrosslinking amino acid to the genetic code of *Escherichia coli*. Proc Natl Acad Sci U S A. 2002;99(17):11020-4. Epub 2002/08/03. doi: 10.1073/pnas.172226299. PubMed PMID: 12154230; PubMed Central PMCID: PMC123203.

4. Datsenko KA, Wanner BL. One-step inactivation of chromosomal genes in *Escherichia coli* K-12 using PCR products. Proc Natl Acad Sci U S A. 2000;97(12):6640-5. Epub 2000/06/01. doi: 10.1073/pnas.120163297. PubMed PMID: 10829079; PubMed Central PMCID: PMC18686.
