# Supplementary material for: Non-pathogenic Escherichia coli acquires virulence by mutating a growth-essential LPS transporter
Source: PLoS Pathog. 2020 Apr 23;16(4):e1008469. doi: 10.1371/journal.ppat.1008469 (PMC7179839; doi:10.1371/journal.ppat.1008469)
Supplement: S4 Table — (DOCX) [file ppat.1008469.s011.docx]

**S4 Table. Primers used in this study**

| Primers to amplify *lptD* or *lptE* | |
| --- | --- |
| lptD-F | ATTTTACGGCTACGGGCTTT |
| lptD-R | GCAGCGTTTTCCAGTTCTTC |
| lptE-F | AAAGTCCGTGCCAAAATCAC |
| lptE-R | TCCAGTCAGTGTTGGGATCA |
|  | |
| Targeting primers to insert chloramphenicol resistant gene | |
| int(insH1-Int)-H1-P1 | CCGGAATACATCCCATATTTCGGCACGCTCACCGGATGCGGTGTAGGCTGGAGCTGCTTC |
| int(insH1-Int)-H2-P2 | TGGATAGGAGAATATTGTTATCCGGATAATGCACTGATGCCATATGAATATCCTCCTTAG |
| int(caiE-F)-H1-P1 | TGCCGGATGCGACCTGGTTGCTGGATAAGATGCTACAACAGTGTAGGCTGGAGCTGCTTC |
| int(caiE-F)-H2-P2 | TTATCTCGTTGATACCGGGCGTCCTGCTTGCCAGATGCGACATATGAATATCCTCCTTAG |
|  |  |
| Targeting ssDNA for suppressor mutations | |
| LptD-G445A | CTGGAACCGACCATCAATTTGCCGCTCTCTAATAATTGGGACTCGATTAATACCGAAGCGAAGTTGCTGGCAACCCATTATCA |
| LptE-6G>A | AAACTCCTCAATCTGGTCGTTGGCTAAGCGCGGGAAGAAGCGTGCGCTACTTAGCAACATTGTTGTTATCTCTGGCGGTGTTAATCAC |
|  | |
| Targeting ssDNA for *lptE* amber mutations | |
| T86am | ATCCTTGCGTTTGGGTAAAGTGAGCATCGCGAAAGACTAGGCCTCGGTATTCCGTAACGGTCAAACAGCAGAGTATCA |
| F90am | GGTAAAGTGAGCATCGCGAAAGATACCGCATCGGTGTAGCGCAATGGTCAAACAGCAGAGTATCAGATGATCATGACGGT |
| R124am | GGCCGTGATATCTACCCGATTAGCGCCAAAGTCTTTTAGAGCTTCTTCGATAACCCGCAAATGGCGTTAGCGAAAGA |
| R150am | AACGAACAAGACATGATCGTAAAAGAGATGTACGATTAGGCCGCCGAACAGCTGATTCGTAAGCTGCCAAGCATCCG |
| T86am-T95I | ATCCTTGCGTTTGGGTAAAGTGAGCATCGCGAAAGACTAGGCCTCGGTATTCCGTAACGGTCAAATAGCAGAGTATCA |
| F90am-T95I | GGTAAAGTGAGCATCGCGAAAGATACCGCATCGGTGTAGCGCAATGGTCAAATAGCAGAGTATCAGATGATCATGACGGT |
|  |  |
| Targeting ssDNA for *lptD* and *lptE* mutations in clinical isolates | |
| LptE-A83S-K84Q | TTCCATCCTTGCGTTTGGGTAAAGTGAGCATCTCACAGGATACCGCATCGGTATTCCGTAACGGTCAAAC |
| LptE-A120T | GTG TTG ATC CCC GGC CGT GAT ATC TAC CCG ATT AGT ACG AAG GTG TTC CGT TCG TTC TTC GAT AAC CCG CAA ATG GCG TT |
| LptE-D141E-V144I | CCG CAA ATG GCG TTA GCG AAA GAT AAC GAA CAA GAA ATG ATT ATT AAA GAG ATG TAC GAC CGT GCT GCC GAA CAG CTG AT |
| LptE-I101V-N105S-T107S | TATTCCGTAACGGTCAAACAGCAGAGTATCAGATGGTGATGACTGTTAGCGCGTCGGTGTTGATCCCCGGCCGTGATATCTACCCGATTAG |
| LptE-K79A | CCACGCGTAAGGACGTTCCATCCTTGCGTTTGGGTGCTGTATCAATCTCACAGGATACCGCATCGGTATTCCGTAACGG |
| LptE-E171K | GTAAGCTGCCAAGCATCCGTGCTGCGGATATTCGTAGTGATAAAGAGCAAACGTCGACCACAACGGATACTCCGGCAACGCCTGC |
| LptE-P181L | TTCGTTCCGACGAAGAACAGACGTCGACCACAACGGACACCCTGGCGACCCCTGCACGCGTCTCCACCACGCTGGGTAACTGATG |
| LptD-Del_K92E93 | GCCGTCTGCAGGCCGACGAAGTGCAGCTCCATCAAGCACCAGGACAACCGGAGCCGGTACGTACCGTTGA |
| LptD-D57N | TACAGGGCGATACCAATGACTTACCCGTGACTATCAACGCGAATCATGCCAAAGGGGACTACCCGGATGACGCCGTGTTTACTGG |
| LptD-P209S | TTGCGGAGATCTGGAACGCCCGCTTTAAGGTGGGTCCGGTTTCCATTTTCTATAGCCCCTATTTGCAGTTGCCGGTGGGTGACAA |
| LptD-E137K | AAGGCTGGGCGAATCTGAACACCAAAGATACCAACGTATGGAAAGGCGACTACCAGATGGTGGGTCGCCAGGGTCGCGGTAAAGC |
| LptD-W249R | ACACCACCACCAACTACTTTGAGTTCTACCTGCCATACTATCGCAATATTGCGCCAAATATGGATGCCACCATCACGCCGCATTA |
| LptD-E299K | GCTTGATGGAACTGGACTATCTGCCTTCAGATAAAGTGTACAAAGACGAGCACCCGAACGATGACAGTTCACGTCGTTGGTTATT |
| LptD-N316Q | GAACACCCGAACGATGACAGTTCACGTCGTTGGTTGTTTTATTGGCAACACTCCGGGGTCATGGATCAGGTGTGGCGTTTCAA |
| LptD-N316K | GAACACCCGAACGATGACAGTTCACGTCGTTGGTTGTTTTATTGGAAACACTCCGGGGTCATGGATCAGGTGTGGCGTTTCAA |
| LptD-K473R-D475A-V478A | CCAATCTTGACTGGTATAACTCCAGAAACACGACCCGCCTGGCGGAATCCGCGAACCGCGTAATGCCGCAATTCAAAGTTGACGG |
| LptD-D475A-V478A | TGACTGGTATAACTCCAGAAACACGACCAAGCTGGCGGAGAGCGCGAACCGCGTAATGCCGCAATTCAAAGTTGACGG |
| LptD-K490R | AAGCTGGACGAATCCGTTAACCGCGTAATGCCGCAGTTCAAGGTAGACGGTAGAATGGTCTTTGAACGCGATATGGAAATGCTGGCTC |
| LptD-D524K | GCGCGCAGTATTTGTACGTGCCGTATCGCGATCAGTCGAAAATCTATAACTACGACTCGTCTCTGCTGCAATCTGA |
| LptD-E618D | GGTTCACTGGTGTGGGCAGGCGATACTTACTGGCGCATTTCTGATCGTTGGGGATTGCGTGGCGGGATTCAGTACGATAC |
|  |  |
| Primers for plasmids overproducing partial LptD or partial LptE | |
| BimpHis2N | GGAGGATCCGGGGGTCATGGATCAGGTG |
| BimpHis2C | AAGAAGCTTCGAACGCAGCATCTCTTG |
| LptE-N2-NcoI | ATATCCATGGGCTGGCATCTGCGT |
| LptE-C-XhoI | ACGTCTCGAGGTTACCCAGCGTGGTGGAGAC |
